# Convalescent plasma in patients admitted to hospital with COVID-19 (RECOVERY): a randomised controlled, open-label, platform trial

**DOI:** 10.1016/S0140-6736(21)00897-7

**Published:** 2021-05-29

**Authors:** RECOVERY Collaborative Group, Obbina Abani, Ali Abbas, Fatima Abbas, Mustafa Abbas, Sadia Abbasi, Hakam Abbass, Alfie Abbott, Nabeel Abdallah, Ashraf Abdelaziz, Mohamed Abdelfattah, Bushra Abdelqader, David Abdo, Basir Abdul, Althaf Abdul Rasheed, Ajibode Abdulakeem, Rezan Abdul-Kadir, Amina Abdulle, Abdulfatahi Abdulmumeen, Rasheed Abdul-Raheem, Niyaz Abdulshukkoor, Kula Abdusamad, Yazeed Abed El Khaleq, Mai Abedalla, Abeer Ul Amna Abeer Ul Amna, Katrina Abernethy, Adebanke Aboaba, Hani Abo-Leyah, Ahmed Abou-Haggar, Mahmoud Abouibrahim, Miriam Abraham, Tizzy Abraham, Abraheem Abraheem, Judith Abrams, Hyacinth-John Abu, Ahmed Abu-Arafeh, Syed M Abubacker, Akata Abung, Yaa Aceampong, Amaka Achara, Devikumar Acharya, Sarah Acheampong, Janet Acheson, Andres Acosta, Catherine Acton, Jacqueline Adabie-Ankrah, Sara Adair, Fiona Adam, Matthew Adam, Huzaifa Adamali, Carol Adams, Charlotte Adams, Kate Adams, Lisa Adams, Richard Adams, Tim Adams, Kirsty Adcock, Jemaimah Addai, Ade Adebiyi, Ken Adegoke, Vicki Adell, Debo Ademokun, Sherna Adenwalla, Oluwasegun A Adesemoye, Emmanuel O Adewunmi, Joyce Adeyemi, Rina Adhikary, Gabrielle Adkins, Adnan Adnan, John Aeron-Thomas, Debbie Affleck, Dominic Affron, Carmel Afnan, Muhammad Afridi, Zainab A Aftab, Meenakshi Agarwal, Rachel Agbeko, Chris Agbo, Penny Agent, Sunil Aggarwal, Arameh Aghababaie, Shafana Ahamed Sadiq, Mohamed H Ahammed Nazeer, Humayun Ahmad, Mohammad Ahmad, Syed Ahmad, Asim Ahmed, Bilal Ahmed, Forizuddin Ahmed, Hamze Ahmed, Iram Ahmed, Irshad Ahmed, Khaled Ahmed, Liban Ahmed, Mahin Ahmed, Maria C Ahmed, Muhammad S Ahmed, Naseer Ahmed, Nausheen Ahmed, Osama Ahmed, Rajia A Ahmed, Rizwan Ahmed, Saif Ahmed, Sammiya Ahmed, Sara Ahmed, Sophia Ahmed, Syed Ahmed, Syed Haris Ahmed, Roa Ahmed Ali, Sana Ahmed, Sana Ahmer, Dhiraj Ail, Mark Ainsworth, Giulia Airoldi, Myriam Aissa, Lindianne Aitken, Bini Ajay, Francis Ajeneye, Abdulakeem Ajibode, Ayesha Ajmi, Tahir Akbar, Naim Akhtar, Nasim Akhtar, Nauman Akhtar, Suha Akili, Oludoyinsola Akindolie, Yinka Akinfenwa, Olugbenga Akinkugbe, Ibrahim Akinpelu, Olajide Akinwumiju, Olugbenro Aktinade, Ahmad Al Aaraj, Asma Al Balushi, Majd Al Dakhola, Aladdin Al Swaifi, Eslam Al-Abadi, Narendra Aladangady, Ayaz Alam, Sajid Alam, Abbas Al-Asadi, Karina Al-Asadi, Kyriaki Alatzoglou, Manaf Al-Bayati, Paul Albert, Lorraine Albon, Gemma Alcorn, Stephen Alcorn, Aggie Aldana, David Alderdice, Rayan Aldouri, Jonathan Aldridge, Nicolas Aldridge, Ana Alegria, Alison Alexander, John Alexander, Peter D G Alexander, Charlotte Alford, Julyan Al-Fori, Laith Alghazawi, Bahij Al-Hakim, Shams Al-Hity, Ali Ali, Asad Ali, Fawzia R Ali, Hoodo Ali, Jawad Ali, Mariam Ali, Mohammad Ali, Nayab Ali, Oudai Ali, Sabira Ali, Sakina Ali, Syed Ali, Abid Alina, Fine Aliyuda, Katrin Alizadeh, Maithem Al-Jibury, Saba Al-Juboori, Majid Al-Khalil, Moutaz Alkhusheh, Allameddine Allameddine, Fiona Allan, Rachel Allan, Alison Allanson, Robert Allcock, Beverley Allen, Eireann Allen, Jess Allen, Kerry Allen, Laura Allen, Louise Allen, Poppy Allen, Rebecca Allen, Sam Allen, Sharon Allen, Simon Allen, Kathryn Allison, Bethan Allman, Lynne Allsop, Hassan Al-Moasseb, Magda Al-Obaidi, Lina Alomari, Akram Al-Rabahi, Bahar Al-Ramadhani, Zayneb Al-Saadi, Inji Alshaer, Rustam Al-Shahi Salman, Warkaq Al-Shamkhani, Bashar Al-Sheklly, Sara Altaf, Mary Alvarez, Balaal Alyas, Maysaa Alzetani, Susan Amamou, Noor Amar, Sakkarai Ambalavanan, Sarah-Jayne Ambler, Robert Ambrogetti, Chris Ambrose, Amir Ameen, Kenneth Amenyah, Maria R Amezaga, Allison Amin, Amina Amin, Kanish Amin, Syed Amin, Tara Amin, Amjad Amjad, Neelma Amjad, Mariam Ammoun, Victoria Amosun, Khaled Amsha, Pugh Amy, Atul Anand, Rekha Anand, Samantha Anandappa, Julie Anderson, Kevin Anderson, Laura Anderson, Michelle Anderson, Nicola Anderson, Rachel Anderson, Rory Anderson, Wendy Anderson, Prematie Andreou, Angela Andrews, Antonette Andrews, Jill Andrews, Susan Andrews, Gregory Andrikopoulos, Kanayochukwu Aneke, Andrew Ang, Wan Wei Ang, Tammy Angel, Aramburo Angela, Paola Angelini, Lazarus Anguvaa, Oleg Anichtchik, Millicent Anim-Somuah, Krishnan Aniruddhan, Jessica Annett, Patrick J Anstey, Rebekah Anstey, Alpha Anthony, Aaron Anthony-Pillai, Philip Antill, Zhelyazkova Antonina, Varghese Anu, Muhammad Anwar, George Apostolides, Aristeidis Apostolopoulos, Sarah Appleby, Diane Appleyard, Maia Far Aquino, Bianca Araba, Samuel Aransiola, Mariana Araujo, Emily Arbon, Ann Archer, Denise Archer, Simon Archer, Christian Ardley, Ana-Maria Arias, Ryoki Arimoto, Charlotte Arkley, Charlotte Armah, Ilianna Armata, Adam Armitage, Ceri Armstrong, Maureen Armstrong, Sonia Armstrong, Sylvia Armstrong-Fisher, Philippa Armtrong, Heike Arndt, Clare Arnison-Newgass, David Arnold, Rachael Arnold, Sarah Arnott, Dhawal Arora, Kavan Arora, Pardeep Arora, Rishi Arora, Andrea Arroyo, Arslam Arter, Ayush Arya, Rita Arya, Denisa Asandei, Adeeba Asghar, Catherine Ashbrook-Raby, Glen Ashby, Helen Ashby, Jan Ashcroft, John Ashcroft, Samuel Ashcroft, Deborah Asher, Ayesha Ashfaq, Ben Ashford, Suhail Ashgar, Abdul Ashish, Sally Ashman-Flavell, Sundar Ashok, Abd-El-Aziz Ashour, Muhammad Z Ashraf, Saima Ashraf, Mohammad B Ashraq, Deborah Ashton, Susan Ashton, Andrew Ashworth, Rebecca Ashworth, Arshia Aslam, Harshini Asogan, Abigail Asquith, Atif Asrar, Omar Assaf, Raine Astin-Chamberlain, Richard Athay-Hunt, Deborah Athorne, Billie Atkins, Christopher Atkins, Stacey Atkins, John Atkinson, Vicki Atkinson, Brygitta Atraskiewicz, Claire Atterbury, Abdul Ahmad Attia, Rita Atugonza, Paula Aubrey, Avinash Aujayeb, Aye Chan Thar Aung, Hnin Aung, Kyaw Thu Aung, Ni Aung, Yin Aung, Zaw Myo Aung, Emily Austin, Karen Austin, Abdusshakur Auwal, Miriam Avery, Joanne Avis, Georgina Aviss, Cristina Avram, Paula Avram, Gabriel Awadzi, Atia Awan, Aszad Aya, Eman Ayaz, Amanda Ayers, Jawwad Azam, Mohammed Azharuddin, Ghazala Aziz, N Aziz, Ali Azkoul, Ashaari Azman Shah, Giada Azzopardi, Hocine Azzoug, Fiyinfoluwa Babatunde, Melvin Babi, Babiker Babiker, Gayna Babington, Matthew Babirecki, Marta Babores, Adetona O Babs-Osibodu, Sammy Bacciarelli, Roudi Bachar, Gina Bacon, Jenny Bacon, Bibi Badal, Gurpreet R Badhan, Shreya Badhrinarayanan, Joseph P Bae, Sibel Bafekr Mishamandani, Alice Baggaley, Amy Baggott, Graham Bagley, Dinesh Bagmane, Lynsey Bagshaw, Kasra Bahadori, James Bailey, Katie Bailey, Lindsey Bailey, Liz Bailey, Morgan Bailey, Pippa Bailey, Sarah Bailey, Stephen Bailey, Hamish Baillie, J Kenneth Baillie, Jennifer Bain, Sanchia Baines, Vikram Bains, Aimi Baird, David Baird, Susan Baird, Tracy Baird, Yolanda Baird, Aiysha Bajandouh, Charles Baker, Emma Baker, Evelyn Baker, Johanne Baker, Josephine Baker, Kenneth Baker, Rebecca Baker, Terri-Anne Baker, Victoria Baker, Hugh Bakere, Nawar Bakerly, Michelle Baker-Moffatt, Nauman Bakhtiar, Panos Bakoulas, Julie Balaam, Niranjan Balachandran, Andrea Balan, Theodosios Balaskas, Madhu Balasubramaniam, Alison Balcombe, Alexander Baldwin, Ashley Baldwin, Caron Baldwin, Danielle Baldwin, Rebekah Baldwin-Jones, James Balfour, Gagan Bali, Sonya Balkee, Ceri Ball, Kasia Ballard, Amy Ballinger, Ismael Balluz, Craig Balmforth, Emese Balogh, Amir Baluwala, Gabby Bambridge, Alasdair Bamford, Amy Bamford, Peter Bamford, Adefunke Bamgboye, Elizabeth Bancroft, Hollie Bancroft, Tanya Bancroft, Joyce Banda, Krishna Bandaru, Srini Bandi, Nageswar Bandla, Somaditya Bandyopadhyam, Amit Banerjee, Millie Banerjee, Ritwik Banerjee, Harrison Banks, Lauren Banks, Luke Banks, Paul Banks, Oliver Bannister, Bharat Bansal, Robert Banthorpe, Laura Banton, Mariamma Baptist, Tanya Baqai, Ananya Mouli Baral, Desislava Baramova, Alex Barber, Russel Barber, Emma Barbon, Miriam Barbosa, Monica Barbosa, Jamie Barbour, Alexander Barclay, Claire Barclay, George Bardsley, Stephanie Bareford, Shahedal Bari, Amy Barker, Debbie Barker, Helen Barker, Joseph Barker, Leon Barker, Oliver Barker, Kerry Barker-Williams, Sinha Barkha, Juliana Barla, Gavin Barlow, Richard Barlow, Valerie Barlow, James Barnacle, Alex Barnard, Debi Barnes, Nicky Barnes, Theresa Barnes, Calum Barnetson, Amy Barnett, Matthew Barnett, Ashton Barnett-Vanes, William Barnsley, Andrew Barr, David Barr, Shaney Barratt, Manuella Barrera, Amy Barrett, Fiona Barrett, Jessica Barrett, Joe Barry, Jazz Bartholomew, Claire Bartlett, Georgina Bartlett, Greg Barton, Jill Barton, Lorna Barton, Rachael Barton, Rosaleen Baruah, Sonia Baryschpolec, Archana Bashyal, Betsy Basker, Ayten Basoglu, Gilda Bass, John Bassett, G Bassett, Chris Bassford, Pavinder Bassi, Betsy Bassis, Bengisu Bassoy, Victoria Bastion, Anupam Basumatary, Tristan Bate, Harry J Bateman, Ian Bateman, Kathryn Bateman, Vhairi Bateman, Eleanor Bates, Hayley Bates, Michelle Bates, Simon Bates, Sally Batham, Ana Batista, Amit Batla, Dushyant Batra, Donna Batty, Harry Batty, Thomas Batty, Peter Baughan, Miranda Baum, Carina Bautista, Fareha Bawa, Fatima S Bawani, Simon Bax, Lydia Baxter, Matt Baxter, Nicola Baxter, Zachary Baxter, Hannah Bayes, Charlotte Baylem, Lee Bayliss, Eileen Bays, Farid Bazari, Rohit Bazaz, Ahmad Bazli, Laura Beacham, Wendy Beadles, Philip Beak, Andy Beale, Kirk Beard, Jack Bearpark, Karen Beaumont, Dawn Beaumont-Jewell, Theresa Beaver, Sarah Beavis, Christy Beazley, Sarah Beck, Virginia Beckett, Rosie Beckitt, Heidi Beddall, Seonaid Beddows, Deborah Beeby, Gail Beech, Michelle Beecroft, Sally Beer, Jane Beety, Gabriela Bega, Alison Begg, Susan Begg, Sara Beghini, Ayesha Begum, Salman Begum, Selina Begum, Teresa Behan, Jasmine Beharry, Roya Behrouzi, Jon Beishon, Claire Beith, James Belcher, Holly Belfield, Katherine Belfield, Ajay Belgaumkar, Dina Bell, Gareth Bell, Gill Bell, Gillian Bell, Joshua Bell, Lauren Bell, Louise Bell, Nicholas Bell, Pippa Bell, Stephanie Bell, Jennifer L Bell, Jennifer Bellamu, Mary Bellamy, Arianna Bellini, Amanda Bellis, Fionn Bellis, Lesley Bendall, Naveena Benesh, Nicola Benetti, Leonie Benham, Guy Benison-Horner, Alexander Bennett, Ann Bennett, Caroline Bennett, Christopher Bennett, Gillian Bennett, Ian Bennett, Kristopher Bennett, Lorraine Bennett, Sara Bennett, Karen Bennion, Vivienne Benson, Jane Benstead, Andrew Bentley, Dionne Bentley, James Bentley, Ian Benton, Eva Beranova, Matthew Beresford, Colin Bergin, Malin Bergstrom, Jolanta Bernatoniene, Thomas Berriman, Zoe Berry, Marnie Berwick, Kimberley Best, Ans-Mari Bester, Yvonne Beuvink, Emily Bevan, Sarah Bevins, Tom Bewick, Andrew Bexley, Sonay Beyatli, Fenella Beynon, Arjun Bhadi, Sanjay Bhagani, Shiv Bhakta, Rekha Bhalla, Khushpreet Bhandal, Kulbinder Bhandal, Ashwin Bhandari, Sangam Bhandari, Aashutosh Bhanot, Ravina Bhanot, Sruti Bhaskara, Prashanth Bhat, Nikhil Bhatia, Rahul Bhatnagar, Karan Bhatt, Janki Bhayani, Deepika Bhojwani, Salimuzzaman Bhuiyan, Anna Bibby, Fatima Bibi, Naheeda Bibi, Salma Bibi, Tihana Bicanic, Sarah Bidgood, Julie Bigg, Sarah Biggs, Alphonsa Biju, Andras Bikov, Sophie Billingham, Jessica Billings, Carron Bilton, Teodirico Binas, Martin Binney, Alice Binns, Muhammad BinRofaie, Oliver Bintcliffe, Catherine Birch, Jenny Birch, Louann Birch, Janet Birchall, Katherine Birchall, Sam Bird, Sumedha Bird, Charndip Biring, Mark Birt, Raquel Bisa, Kilanalei Bishop, Linda Bishop, Lisa Bishop, Karen Bisnauthsing, Nibedan Biswas, Sahar Biuk, Karen Blachford, Ethel Black, Helen Black, Karen Black, Mairead Black, Polly Black, Hayley Blackgrove, Bethan Blackledge, Joanne Blackler, Samantha Blackley, Helen Blackman, Stuart Blackmore, Caroline Blackstock, Loraine Blackwood, Francesca Blakemore, Helen Blamey, Alison Bland, Sujata Blane, Simon Blankley, Mary Blanton, Parry Blaxill, Jenny Blaxland, Katie Blaylock, Jane Blazeby, Carol Blears, Natalie Blencowe, Donna Blofield, Ben Bloom, Jack Bloomfield, Angela Bloss, Hannah Bloxham, Louise Blundell, Andrew Blunsum, Mark Blunt, Ian Blyth, Kevin Blyth, Andrew Blythe, Karen Blythe, Marilyn Boampoaa, Boniface Bobie, Karen Bobruk, Pritesh Bodalia, Neena Bodasing, Tanya Bodenham, Sherin Bodh, Gabriele Boehmer, Marta Boffito, Kristyna Bohmova, Sumit Bokhandi, Maria Bokhar, Saba Bokhari, Sakina Bokhari, Syed Owais Bokhari, Ambrose Boles, Sarah Bollington, Sam Bolton, Charlotte Bomken, Charlotte Bond, Hayley Bond, Helena Bond, Stuart Bond, Thomas Bond, Alice Bone, Georgia Boniface, Wendy Bonnert, Lizzy Bonney, Leigh Boorman, Catherine Booth, Joanne Borbone, Cameron Borhani, Naomi Borman, Rachel Borrell, Mamu Boshir, Fiona Bottrill, Laura Bough, Hayley Boughton, Zoe Boult, Miriam Bourke, Stephen Bourke, Michelle Bourne, Rachel Bousfield, Lucy Boustred, Darren Bowen, Kaye Bowen, Alexandra Bowes, Amy Bowes, Philip Bowker, Louise Bowman, Simon Bowman, Rachel Bowmer, Angie Bowring, Geoff Bowyer, Helen Bowyer, Carmel Boyd, Jenny Boyd, Laura Boyd, Maxine Boyd, Namoi Boyle, Pauline Boyle, Rosalind Boyle, Louise Boyles, Osman Bozdag, Leanna Brace, David Brack, Charlotte Brackstone, Rob Bradburn, Jodie Bradder, Barry Bradley, Clare Jane Bradley, Pamela Bradley, Patrick Bradley, Paul Bradley, Joanne Bradley-Potts, Lynne Bradshaw, Sarah Bradshaw, Zena Bradshaw, Rebecca Brady, Shirin Brady, Denise Braganza, Suhail Brailsford, Jill Braithwaite, Marie Branch, Thomas Brankin-Frisby, Jamie Brannigan, Louise Brassington, Sophie Brattan, Fiona Bray, Nancy Bray, Angela Brazier, Manny Brazil, Lucy Brear, Tracy Brear, Stephen Brearey, Laura Bremner, Morwenna Brend, Giovanna Bretland, Chris Brewer, Hannah Bridge, Gavin Bridgwood, Hayley Briggs, Mark Briggs, Sara Brigham, John Bright, Chris Brightling, Lutece Brimfield, Elaine Brinkworth, Robin Brittain-Long, Vianne Britten, Terri Brittin, Lauren Broad, Sarah Broad, Rosie Broadhurst, Andrew Broadley, Marie Broadway, Christopher Brockelsby, Megan Brocken, Tomos Brockley, Andrew Broderick, Mary Brodsky, Fiona Brogan, David Broggio, Liz Brohan, Felicity Brokke, Jacob Brolly, David Bromley, Hannah Brooke-Ball, Verity Brooker, Ceri Brookes, Matthew Brookes, Alison Brooks, Karen Brooks, Nicole Brooks, Philip Brooks, Rachel Brooks, Sophie Brooks, Natalie Broomhead, Chloe Broughton, Nathaniel Broughton, Matt Brouns, Marie Browett, Alison Brown, Ammani Brown, Carly Brown, Catrin Brown, Chloe Brown, Elizabeth Brown, Ellen Brown, Heather Brown, Janet Brown, Louise Brown, Niall Brown, Pauline Brown, Rachel Brown, Richard Brown, Robert Brown, Steven Brown, Tom Brown, Wayne Brown, Bria Browne, Charlotte Browne, Duncan Browne, Mitchell Browne, Stephen Brownlee, Alba Brraka, David Bruce, Johanna Bruce, Michelle Bruce, Wojciech Brudlo, Nigel Brunskill, Alan Brunton, Margaret Brunton, Mandy Bryan, Meera Bryant, April Buazon, Maya Buch, Julie Buchan, Ruaridh Buchan, Alexis Buchanan, Ruaridh Buchanan, Danielle Buche, Amanda Buck, Matthew Buckland, Laura Buckley, Philip Buckley, Sarah Buckley, Carol Buckman, Kathleen Buckmire, George Bugg, Ramadan Bujazia, Marwan Bukhari, Shanze Bukhari, Richard Bulbulia, Alex Bull, Damian Bull, Rhian Bull, Thomas Bull, Naomi Bulteel, Kasun Bumunarachchi, Roneleeh Bungue-Tuble, Caroline Burchett, Dorota Burda, Christy Burden, Thomas G Burden, Mika Burgess, Paula Burgess, Richard Burgess, Sophia Burgess, Paula Burgett, Adrian Burman, Sara Burnard, Caroline Burnett, John Burnett, Amanda Burns, Amy Burns, Collette Burns, James Burns, Karen Burns, Samuel Burns, Sarah Burns, Daniel Burrage, Sadie Burrage, Kate Burrows, Claire Burston, Ben Burton, Fiona Burton, Matthew Burton, Angela Busby, Deborah Butcher, Aaron Butler, Jessica Butler, Joanne Butler, Joshua Butler, Lesley Butler, Peter Butler, Susan Butler, Al-Tahoor Butt, Mohammad M Butt, Sophia Butt, Caryl Butterworth, Nicola Butterworth-Cowin, Robert Buttery, Tom Buttle, Heather Button, Daniel Buttress, Jane Byrne, Wendy Byrne, Victoria Byrne-Watts, Eleanor Byworth, Amanda Cabandugama, Ruth Cade, Anthony Cadwgan, Donna Cairney, James Calderwood, Darren Caldow, Moira Caldwell, Giorgio Calisti, Debbie Callaghan, Jennifer Callaghan, Claire Callens, Donaldson Callum, Caroline Calver, Melissa Cambell-Kelly, Tracey Camburn, David R Cameron, Eleanor Cameron, Fraser Cameron, Sarah Cameron, Sheena Cameron, Christian Camm, Renee F D Cammack, Alison Campbell, Amy Campbell, Barbara Campbell, Bridget Campbell, Debbie Campbell, Helen Campbell, Hilary Campbell, Jonathan Campbell, Mark Campbell, Robyn Campbell, Wynny Campbell, Quentin Campbell Hewson, Julie Camsooksai, Ana Canabarro, Lisa Canclini, Shaula Mae Candido, Janie Candlish, Cielito Caneja, Johnathon Cann, Ruby Cannan, Emma Cannon, Michael Cannon, Petra Cannon, Vivienne Cannons, Jane Cantliff, Ben Caplin, Santino Capocci, Noemi Caponi, Angelika Capp, Anne Capps-Jenner, Thomas Capstick, Ishmael Carboo, Nuria Cardenas, Mary Cardwell, Rachel Carey, Simon Carley, Tammy Carlin, Andrew Carlton, Samantha Carmichael, Mandy Carnahan, Rebecca Carnegie, Charlotte Caroline, Emily Carpenter, Jodi Carpenter, David Carr, Sharon Carr, Anna Carrasco, Samantha Carrington, Zoe Carrington, Paul Carroll, Caroline Carron, Anne Carstairs, Jonathan Carter, Michael Carter, Moira Carter, Paul Carter, Penny Carter, Steven Carter, Simon Carter-Graham, Douglas Cartwright, Jo-Anne Cartwright, Claire Carty, Sinead Carty, Jaime Carungcong, Carolyn Carveth-Marshall, Susan Casey, Annie Cassells, Barbara Cassimon, Teresa Castiello, Gail Castle, Bridget Castles, Melanie Caswell, Ana Maria Catana, Heidi Cate, Susanne Cathcart, Katrina Cathie, Christine Catley, Laura Catlow, Matthew Caudwell, Jill Caulfield, Anna Cavazza, Luke Cave, Simon Cavinato, Frianne Cawa, Kathryn Cawley, Chloe Caws, Hankins Cendl, Hannah Century, Jeva Cernova, Mansur Cesay, Ed Cetti, Stephanie Chabane, Manish Chablani, Cathleen Chabo, David Chadwick, Julie Chadwick, Robert Chadwick, Ela Chakkarapani, Arup Chakraborty, Mallinath Chakraborty, Mollika Chakravorty, James Chalmers, Richard Chalmers, Georgina Chamberlain, Sarah Chamberlain, Emma Chambers, Jonathan Chambers, Lucy Chambers, Naomi Chambers, Alex Chan, Carmen Chan, Cheuk Chan, Evelyn Chan, Kayen Chan, Kimberley Chan, Ping Chan, Rebekah (Pui-Ching) Chan, Xin Hui Chan, Chris Chandler, Heidi Chandler, Kim J Chandler, Stuart Chandler, Zoe Chandler, Vikki Chandler-Vizard, Sumit Chandra, Navin Chandran, Badrinathan Chandrasekaran, Cherry Chang, Yvonne Chang, Josephine Chaplin, Graeme Chapman, John Chapman, Katie Chapman, Laura Chapman, Lianne Chapman, Polly Chapman, Timothy Chapman, Lucy Chappell, Linda Chapple, Amanda Charalambou, Bethan Charles, Dianne Charlton, Sally Charlton, Kevin Chatar, Calvin Chatha, Ritesh Chaube, Muhammad YN Chaudhary, Iram Chaudhry, Nazia Chaudhuri, Muhammad Chaudhury, Anoop Chauhan, Ruchi S Chauhan, Vipul Chauhan, Nicola Chavasse, Iknam Chaven, Rosanna Chavez, Vipal Chawla, Maria Cheadle, Lindsay Cheater, James Cheaveau, Charlotte Cheeld, Michelle Cheeseman, Fang Chen, Hui Min Chen, Terence Chen, Lok Yin Cheng, Zhihang Cheng, Helen Chenoweth, Chun How Cheong, Shiney Cherian, Suzanne Cherif, Mary Cherrie, Helen Cheshire, Barry Chesterson, Betty Cheung, Chee Kay Cheung, Elaine Cheung, Michelle Cheung, Claire Cheyne, Swati Chhabra, Wei Ling Chia, Eric Chiang, Angela Chiapparino, Rosavic Chicano, Zviedzo A Chikwanha, Sam Chilcott, Phillipa Chimbo, KokWai Chin, Wen Jie Chin, Rumbidzai Chineka, Amol Chingale, Karen Chinn, Vashira Chiroma, Heather Chisem, Claire Chisenga, Ben Chisnall, Carolyn Chiswick, Sunder Chita, Nihil Chitalia, Matthew Chiu, Brenda Chivima, Catherine Chmiel, Soha Choi, Willy Choon Kon Yune, Vandana Choudhary, Sarah Choudhury, Bing-Lun Chow, Fateha Chowdhury, Mahibbur Chowdhury, Shahid Chowdhury, Victoria Christenssen, Peter Christian, Alexander Christides, Fiona Christie, Daniel Christmas, Thereza Christopherson, Mark Christy, Paris Chrysostomou, Yunli Chua, Shabs Chucha, Dip Chudgar, Richard Chudleigh, Srikanth Chukkambotla, Michael E Chukwu, Izu Chukwulobelu, Chi Y Chung, Ben Church, Elaine Church, Sara R Church, David Churchill, Nicole Cianci, Nick Ciccone, Paola Cicconi, Paola Cinardo, Zdenka Cipinova, Bessie Cipriano, Sarah Clamp, Melanie Clapham, Edel Clare, Sarbjit Clare, Andrew Clark, Charlotte Clark, Diane Clark, Felicity Clark, Gabrielle Clark, James Clark, Katherine Clark, Kaylea Clark, Louise Clark, Lucy Clark, Matthew Clark, Patricia Clark, Richard Clark, Thomas Clark, Wendy Clark, Zoe Clark, Andrea Clarke, Heather Clarke, Mark Clarke, Paul Clarke, Robert Clarke, Roseanne Clarke, Samantha Clarke, Sarah Clarke, Sheron Clarke, Tracy Clarke, Andrew Clarkson, Alleyna Claxton, Kate Clay, Elizabeth Clayton, Olivia Clayton, Jill Clayton-Smith, Chris Cleaver, Carlota Clemente de la Torre, Jayne Clements, Suzanne Clements, Francesca Clemons, Lee Clifford, Lynne Clifford, Rachael Clifford, Sarah Clifford, Amelia Clive, Jonathan Clouston, Samantha Clueit, Andrea Clyne, Michelle Coakley, Peter G L Coakley, Tony Coates, Kathryn Cobain, Alexandra Cochrane, Patricia Cochrane, Maeve Cockerell, Helen Cockerill, Shirley Cocks, Rachel Codling, Adam Coe, Samantha Coetzee, David Coey, Danielle Cohen, Jonathan Cohen, Oliver Cohen, Mike Cohn, Louise Coke, Olutoyin Coker, Nicholas Colbeck, Roghan Colbert, Carol Cole, Esther Cole, Jade Cole, Joby Cole, Julie Cole, Richard Cole, Sue Cole, Garry Coleman, Matt Coleman, Holly Coles, Rebecca Coles, Macleod Colin, Alicia Colino-Acevedo, Julie Colley, Dawn Collier, Heather Collier, Paul Collini, Emma Collins, Jaimie Collins, Joanne Collins, Nicola Collins, Sally Collins, Vicky Collins, Andrew Collinson, Bernadette Collinson, Jennifer Collinson, Matthew Collis, Madeleine Colmar, Hayley E Colton, James Colton, Katie Colville, Carolyn Colvin, Ryan Colwell, Edward Combes, David Comer, Alison Comerford, Mike Comery, Dónal Concannon, Robin Condliffe, Lynne Connell, Natalie Connell, Karen Connelly, Gavin Connolly, Mireille Connolly, Emma Connor, Samantha Conran, Antonia Conroy, Veronica Conteh, Rory Convery, Camilla Conway, Francesca Conway, Grainne Conway, Rhiannon Conway, Jo-Anna Conyngham, Colette Cook, Eloise Cook, Gemma Cook, Helen Cook, Julie Cook, Danielle Cooke, Graham Cooke, Katrina Cooke, Soo Cooke, Tim Cooke, Adele Cooper, Alison Cooper, Chris Cooper, David Cooper, Helen Cooper, Jamie Cooper, Joanne Cooper, Joshua Cooper, Laura Cooper, Lauren Cooper, Nick Cooper, Rowena Cooper, Emma Cooperwaite, Thomas Cope, Sinead Corbet, Carolyn Corbett, John Corcoran, Chris Cordell, Jessica Cordle, Alasdair Corfield, John Corless, Alison Corlett, Joe Cornwell, Michael Cornwell, Diana Corogeanu, Mirella Corredera, Ruth Corrigan, Catherine Corry, Rita Corser, Denise Cosgrove, Tracey Cosier, Patricia Costa, Charlie Coston, Susannah Cotgrove, Zoe Coton, Lisa-Jayne Cottam, Rhiannon Cotter, Donna Cotterill, Alice Cotton, Caroline Cotton, Katy Cotton, Andrew Coull, James Coulson, David Counsell, David Counter, Cherry Coupland, Ellie Courtney, Julia Courtney, Rebecca Cousins, Alexander Cowan, Elena Cowan, Ruth Cowburn, Richard Cowell, Louise Cowen, Steve Cowman, Amanda Cowton, Debra Anne Cox, Ellie Cox, Giles Cox, Karina Cox, Miriam Cox, Karen Coy, Beverly Craig, Victoria Craig, Felicity Craighead, Matthew Cramp, Beverley Crane, Jacolene Crause, Adrian Crawford, Angie Crawford, Emma Crawford, Isobel Crawford, Richard Crawforth, Sarah Crawshaw, Ben Creagh-Brown, Andrew Creamer, Ryan Creighton, Joanne Cremona, Saveria Cremona, Janet Cresswell, Mark Cribb, Charles Crichton, Declan Crilly, Lauren Crisp, Nikki Crisp, Dominic Crocombe, Maria Croft, Ian Cron, Derrick Crook, Jennifer Crooks, Helen Croot, Harriet Crosby, Sarah Cross, Tim Cross, Amy Crothers, Stephen Crotty, Susan Crouch, Madeleine Crow, Amanda Crowder, Kate Crowley, Teresa Crowley, Rebecca Croysdill, Callum Cruickshank, Conor Cruickshank, Irena Cruickshank, James Cruise, Helen Crumley, Carina Cruz, Trino Cruz Cervera, Dominic Cryans, Guanguo Cui, Helen Cui, Donna Cullen, Lorraine Cullen, Gillian Cummings-Fosong, Marie Cundall, Victoria Cunliffe, Lorraine Cunningham, Neil Cunningham, Nicola Cunningham, Jason Cupitt, Hollie Curgenven, Debra Curley, Gerens Curnow, David Curran, Simon Curran, Craig Currie, Jacqueline Currie, Scarlett Currie, Abi Curtis, Becca Curtis, Jonathan Curtis, Katrina Curtis, Olivia Curtis, Thomas Curtis, Rebecca Cuthbertson, Sean Cutler, Marta Czekaj, Patrycja Czylok, Joana da Rocha, Andrew Dagens, Helen Daggett, Phil Daggett, Jacqui Daglish, Sandeep Dahiya, Helen Dakers-Black, Anne Dale, Katie Dale, Michaela Dale, Sam Dale, Jolyon Dales, Helen Dalgleish, Nikki Dallas, Helen Dallow, Dermot Dalton, Zoe Daly, Akila Danga, Amelia Daniel, Priya Daniel, Allison Daniels, Adela Dann, Sandra Danso-Bamfo, Nimo Daoud, Alex Darbyshire, Janet Darbyshire, Paul Dargan, Paul Dark, Tace Darling, Kate Darlington, Tom Darton, Guledew Darylile, Manjusha Das, Sukamal Das, Martin Daschel, Joanne Dasgin, Dibyendu Datta, Anna Daunt, Emily Davenport, Mark Davey, Michelle Davey, Miriam Davey, Molly Davey, Arlene David, Mini David, Alexander Davidson, Laura Davidson, Neil Davidson, Richard Davidson, Albert Davies, Alison Davies, Amanda Davies, Amy Davies, Angela Davies, Carolyn Davies, Catrin Davies, Cheryl Davies, Drew Davies, Elaine Davies, Ffyon Davies, Helen Davies, James Davies, Jane Davies, Jeni Davies, Jim Davies, Karen Davies, Kelly Davies, Kim Davies, Louisa Davies, Mark Davies, Matthew Davies, Michelle Davies, Nina Davies, Owen Davies, Patrick Davies, Rachel Davies, Rhys Davies, Ruth Davies, Sarah Davies, Simon Davies, Alison Davis, Gwyneth Davis, Illinos Davis, Julie-Ann Davis, Katherine Davis, Peter Davis, Alexander Davison, Sophia Davison, Mark Davy, Christine Dawe, H Dawe, Mark Dawkins, Danielle Dawson, Elizabeth Dawson, Joy Dawson, Susan Dawson, Tom Dawson, Andrew Daxter, Andrew Day, Helena Day, Jacob Day, Jeremy Day, Lynn Day, Jamie D'Costa, Parijat De, Duneesha de Fonseka, Toni de Freitas, Frederico De Santana Miranda, Eleanor de Sausmarez, Shanika de Silva, Thushan de Silva, Jessica De Sousa, Paulo De Sousa, James de Souza, Anthony de Soyza, Natasha de Vere, Johannes de Vos, Bethan Deacon, Sharon Dealing, Anna Dean, Julie Dean, Katrina Dean, Stephen Dean, Tessa Dean, Jill Deane, James Dear, Effie Dearden, Alison Deary, Catherine Deas, Samuel Debbie, Gabor Debreceni, Vashist Deelchand, Matthew Deeley, Joanne Deery, Emmanuel Defever, Raji Dehulia, Max Deighton, Manuela Del Forno, Arnold Dela Rosa, Lisa Delaney, Amanda Dell, Carrie Demetriou, David DeMets, Jane Democratis, Jacqueline Denham, Emmanuelle Denis, Laura Denley, Craig Denmade, Kathy Dent, Martin Dent, Elise Denton, Tom Denwood, Nishigandh Deole, Darshita Depala, Maria Depante, Randle Derbyshire, Susan Dermody, Amisha Desai, Asmita Desai, Purav Desai, Sanjeev Deshpande, Vai Deshpande, Brendan Devine, Sirjana Devkota, Nicola Dewland, Prakash Dey, Vishal Dey, Rogin Deylami, Jazz Dhaliwal, Kevin Dhaliwal, Jas Dhalliwal, Mandeep Dhanda, Sundip Dhani, Amandeep Dhanoa, Mili Dhar, Devesh Dhasmana, Aman Dhesi, Ekanjali Dhillon, Reiss Dhillon, Pamela Diamond, Priya Dias, Stephanie Diaz, Kayleigh Diaz-Pratt, Debbie Dickerson, Pamela Dicks, Stuart Dickson, Julie Dijo, Sean Dillane, Sarah Diment, Paul Dimitri, Maria Dineen, ThaiHa Dinh, Tri Dinh, Alex Dipper, Laura Dirmantaite, Lisa Ditchfield, Sarah Diver, Lavanya Diwakar, Caroline Dixon, Giles Dixon, Stephen Dixon-Mould, Brice Djeugam, Petr Dlouhy, Laurence Dobbie, Marinela Dobranszky Oroian, Charlotte Dobson, Lee Dobson, Marie Docherty, David Dockrell, James Dodd, Jackie Dodds, Rebecca Dodds, Steve Dodds, Richi Dogra, Erin Doherty, Warren Doherty, Yumiko Doi, Iain Doig, Eleanor Doke, Daniel Dolan, Mark Dolman, Rozzie Dolman, Lisa Donald, Callum Donaldson, Christopher Donaldson, Denise Donaldson, Gillian Donaldson, Kate Donaldson, Joanne Donnachie, Christopher Donnelly, Eilish Donnelly, Ronan Donnelly, Aravindhan Donohoe, Gemma Donohoe, Bryan Donohue, Sinead Donton, Emma Dooks, Grainne Doran, Kane Dorey, Sharon Dorgan, Amanda Dornan, Moonira Dosani, Davinder Dosanjh, Paula Dospinescu, Katie Douglas, Jonathan Douse, Lucy Dowden, Michelle Dower, Kerry Dowling, Sud Dowling, Nicola Downer, Charlotte Downes, Rob Downes, Thomas Downes, Damian Downey, Philippa Downey, Robert Downey, Louise Downs, Simon Dowson, Cornel Dragan, Cristina Dragomir, Cristina Dragos, Maire Drain, Chelsea Drake, Victoria Drew, Olivia Drewett, Celine Driscoll, Helena Drogan, Nikki Drogman, Graham Drummond, Ronald Druyeh, Simon Drysdale, An Du Thinh, Hazel Dube, Judith Dube, Ophias Dube, Stephen Duberley, Hayley Duckles-Leech, Nicola Duff, Emma Duffield, Sam Duff-Miller, Helen Duffy, Lionel Dufour, Annette Duggan, Helen Duggan, Parveen Dugh, Janice Duignan, Mick Duley, Simon Dummer, Andrew Duncan, Christopher Duncan, Fullerton Duncan, Gregory Duncan, Stephanie Dundas, Alessia Dunn, Charlotte Dunn, Damian Dunn, Laura Dunn, Paul Dunn, Charlene Dunne, Karen Dunne, Fiona Dunning, Aidan Dunphy, Venkat Duraiswamy, Beatriz Duran, Ingrid DuRand, Steve Durgacharan, Natalie Duric, Alison Durie, Emily Durie, Hannah Durrington, Haris Duvnjak, Akshay Dwarakanath, Laasya Dwarakanath, Ellen Dwyer, Claudia Dyball, Lee Dyble, Kristyn Dyer, Harvey Dymond, Tom Dymond, Chris Eades, Laura Eagles, Joanne Early, Melissa Earwaker, Nicholas Easom, Clare East, Kim East, Amy Easthope, Fraser Easton, Caroline Eaton, Caroline Eaton-Howell, Ruth Eatough, Adrian Ebbs, Oluwadamilola Ebigbola, Daniel Ebner, Martin Ebon, Sinan Eccles, Chloe Eddings, Michael Eddleston, Maureen Edgar, Katharine Edgerley, Nicholas Edmond, Julie Edmonds, Dave Edmondson, Mary Edmondson, Tracy Edmunds, Alexandra Edwards, Catherine Edwards, Joy Edwards, Kennedy Edwards, Mandy Edwards, Tomos Edwards, Jenny Eedle, Dawn Egginton, Loveth Ehiorobo, Sarah Eisen, Ugochukwu Ekeowa, Mohamed Ekoi, Ayomide Ekunola, Soha El Behery, Moulod El-Agnaf, Mohamed Elbeshy, Kate El-Bouzidi, Jennifer Elder, Mohammed El-Din, Diana Eleanor, Ibrahim Eletu, Eman Elfar, Mayy Magdy Elgamal, Amr Elgohary, Stellios Elia, Jennifer Elias, Tania Elias, Nadia Elkaram, Mohammed El-Karim, Andrew Victor Elkins, Julie Ellam, Nikki Ellard, Laura Nicola Ellerton, Lucy Elliot, Amy Elliott, Chris Elliott, Fiona Elliott, Kerry Elliott, Scott Elliott, Toby Elliott, Annie Ellis, Ann-Marie Ellis, Christine Ellis, Kay Ellis, Kaytie Ellis, Tak-Yan Ellis, Yvette Ellis, Megan Ellison, Rahma Elmahdi, Einas Elmahi, Hannah-May Elmasry, Mohammed Elmi, Najla Elndari, Omer Elneima, Mohamed Elokl, Ahmed Elradi, Mohamed Elsaadany, Sally El-Sayeh, Hana El-Sbahi, Tarek Elsefi, Karim El-Shakankery, Robert Elshaw, Hosni El-Taweel, Sarah Elyoussfi, Jonathan Emberey, Jonathan R Emberson, John Emberton, Julian Emmanuel, Ingrid Emmerson, Michael Emms, Florence Emond, Marieke Emonts, Nicu Enachi, Angila Engden, Katy English, Emma Entwistle, Hene Enyi, Marios Erotocritou, Helen Escreet, Peter Eskander, Hanif Esmail, Lise Estcourt, Amy Evans, Brynach Evans, Chris Evans, Daren Evans, Debra Evans, Gail Evans, Gareth Evans, Jennifer Evans, Lisa Evans, Lynn Evans, Margaret Evans, Michelle Evans, Mim Evans, Morgan Evans, Ranoromanana Evans, Ryan Evans, Teriann Evans, Terry John Evans, Tony Eve, Caroline Everden, Serenydd Everden, Hayley Evison, Lynsey Evison, Penny Eyton-Jones, Jacqueline Faccenda, Leila Fahel, Youstina Fahmay, Sara Fairbairn, Terry Fairbairn, Andy Fairclough, Louise Fairlie, Mark Fairweather, Anne Fajardo, Naomi Falcone, Euan Falconer, Jonathan Falconer, John Fallon, Andrea Fallow, David Faluyi, Victoria Fancois, Qayyum Farah, Novin Fard, Amr Farg, Margaret Farinto, Adam Farmer, Katie Farmer, Toni Farmery, Samantha Farnworth, Faiyaz Farook, Hadia Farooq, Sidrah Farooq, Fiona Farquhar, Karen Farrar, Aaron Farrell, Barbara Farrell, James Farthing, Syeda Farzana, Rahmatu Fasina, Azam Fatemi, Mina Fatemi, Nibah Fatimah, Maria Faulkner, Saul N Faust, Joe Fawke, Sinmidele Fawohunre, Abul Fazal, Kelly Feane, Simon Fearby, Alex Feben, Federico Fedel, Daria Fedorova, James Feely-Henderson, Christopher Fegan, Mae Felongco, Lynsey Felton, Tim Felton, Kate Fenlon, Andrea Fenn, Isabelle Fenner, Ciara Fenton, Melisa Fenton, Cameron Ferguson, Jenny Ferguson, Kathryn Ferguson, Katie Ferguson, Stephanie Ferguson, Susan Ferguson, Susie Ferguson, Victoria Ferguson, Denzil Fernandes, Candida Fernandez, Eduardo Fernandez, Maria Fernandez, Sonia Fernandez Lopez, Callum Jeevan Fernando, Ahmed Feroz, Pietro Ferranti, Thais Ferrari Gersogamo, Eleanor Ferrelly, Alexandra Ferrera, Emma Ferriman, Nicholas Fethers, Ben Field, Janet Field, Rebecca Field, Karen Fielder, Lindsey Fieldhouse, Andra Fielding, Julie Fielding, Len Fielding, Sarah Fielding, Asma Fikree, Sarah Ann Filson, Sarah Finbow, Debbie Finch, Joanne Finch, Laurie Finch, Joanne Finden, Natalie Fineman, Lauren Finlayson, Adam Finn, Joanne Finn, Clare Finney, Sofia Fiouni, Jo Fiquet, Dani Fisher, Emily Fisher, James Fisher, Neil Fisher, Meadow Fisher Crisp, Daniel Fishman, Krystofer Fishwick, Lorraine Fitzgerald, Chloe Fitzpatrick-Creamer, Jan Flaherty, Michael Flanagan, Charles Flanders, Cathy Flatters, Julie Fleming, Lucy Fleming, Paul Fleming, William Flesher, Alison Fletcher, Jonathan Fletcher, Lucy Fletcher, Simon Fletcher, Sophie Fletcher, Karen Flewitt, Christopher Flood, Ian Floodgate, Vincent Florence, Sharon Floyd, Kelly Flynn, Rachel Flynn, Sara Flynn, Claire Foden, Adama Fofana, Georgina Fogarty, Claire Foley, Paul Foley, Linda Folkes, Daniela Mock Font, Evodian Fonyonga, Aiwyne Foo, Jane Foo, Andrew Foot, Jayne Foot, Jane Forbes, Jamie Ford, Kathy Ford, Jennifer Foreman, Caroline Fornolles, Adam Forrest, Ellie Forsey, Miranda Forsey, Thomas Forshall, Elliot Forster, Julian Forton, Emily Foster, Joseph Foster, Rachel A Foster, Tracy Foster, Theodora Foukanelli, Angela Foulds, Ian Foulds, Folakemi Fowe, Emily Fowler, Robert Fowler, Stephen Fowler, Caroline Fox, Claire Fox, Daniel Fox, Heather Fox, Jonathan Fox, Lauren Fox, Natalie Fox, Olivia Fox, Simon Fox, Sarah-Jane Foxton, Yasin Fozdar, Rebecca Frake, Alex Francioni, Olesya Francis, Rebecca Francis, Sarah Francis, Theodora Francis-Bacon, Jason Frankcam, Helen Frankland, Gayle Franklin, Jessica Franklin, Darron Franks, Catherine Fraser, Laura Fraser, Sharon Frayling, Martyn Fredlund, Matthew Free, Carol Freeman, Elaine Freeman, Hannah Freeman, Nicola Freeman, Clare Freer, Ian Freestone, Eleanor French, Matthew Frise, Renate Fromson, Claire Froneman, Adam Frosh, John Frost, Victoria Frost, Oliver Froud, Rachel Frowd, Arun Fryatt, Jake Fryer, Janet Fu, Bridget Fuller, Liz Fuller, Neil Fuller, Tracy Fuller, Duncan Fullerton, Jenny Fullthorpe, Carrie Fung, Gayle Fung, Sarah Funnell, John Furness, Charlene Furtado, Andrew Fyfe, Nytianandan G, Elizabeth Gabbitas, Claire Gabriel, Diana Gabriel, Hadiza Gachi, Rose Gad, Joshua Gahir, Sarveen Gajebasia, Katarzyna Gajewska-Knapik, Zacharoula Galani, Christopher Gale, Hugo Gale, Rebecca Gale, Swetha Gali, Karen Galilee, Bernadette Gallagher, Jude Gallagher, Rosie Gallagher, William Gallagher, Joanne Galliford, Catherine Galloway, Chris Galloway, Emma Galloway, Jacqui Galloway, James Galloway, Laura Gamble, Liz Gamble, Brian Gammon, Jaikumar Ganapathi, Ramesh Ganapathy, Kaminiben Gandhi, Sarah Gandhi, Usha Ganesh, Abrar Gani, Emma-James Garden, Antoni D Gardener, Emma Gardiner, Jill Gardiner, Michael Gardiner, Phil Gardiner, Siobhan Gardiner, Caroline Gardiner-Hill, Jonathan Gardner, Mark Garfield, Atul Garg, Nathan Garlick, Justin Garner, Lucie Garner, Zoe Garner, Kimberley Garnett, Robert Garney, Rosaline Garr, Michael Garstka, Peter Gartan, Florence Garty, Rachel Gascoyne, Hyeriju Gashau, Noha Gasmalseed, Michaela Gaspar, Aoife Gatenby, Erin Gaughan, Alok Gaurav, Mariana Gavrila, Jane Gaylard, Emma Gaywood, Catherine Geddie, Alison Geddis, Ian Gedge, Sarah Gee, Minerva Gellamucho, Karzan Gelly, Leila Gelmon, Sandra Gelves-Zapata, Gemma Genato, Susan Gent, Natalie Geoghegan, Chloe George, Sam George, Tina George, Simon Georges, Domonique Georgiou, Peter Gerard, Leigh Gerdes, Louise Germain, Helen Gerrish, Abel Getachew, Louise Gethin, Hisham Ghanayem, Amardeep Ghattaoraya, Anca Gherman, Alison Ghosh, Justin Ghosh, Sudhamay Ghosh, Sarra Giannopoulou, Malick Gibani, Andrew Gibb, Ben Gibbison, Kerry Gibbons, Alex Gibson, Bethan Gibson, Kimberley Gibson, Kirsty Gibson, Sian Gibson, Cat Gilbert, Jeanette Gilbert, Joanne Gilbert, Kayleigh Gilbert, Sean Gilchrist, Benjamin Giles, Mandy Gill, Rose Gill, Lynne Gill, Paul Gillen, Annelies Gillesen, Katherine Gillespie, Matt Gillespie, Elizabeth Gillham, Andrew Gillian, Deborah Gilliland, Robert Gillott, Danielle Gilmour, Kate Gilmour, Theodora Giokanini-Royal, Anna Gipson, Joanna Girling, Rhian Gisby, Angelena Gkioni, Aikaterini Gkoritsa, Effrossyni Gkrania-Klotsas, Amy Gladwell, James Glanville, Jessica Glasgow, Susannah Glasgow, Jon Glass, Lynn Glass, Sharon Glaysher, Lisa Gledhill, Ana Glennon, John Glover, Kyle Glover, Jan Glover Bengtsson, Chevanthy Gnanalingam, Julie Goddard, Wendy Goddard, Emily Godden, Jo Godden, Gillian Godding, Emma Godson, Gerry Gogarty, Sukanya Gogoi, Aiky Goh, Rebeca Goiriz, Sriya Gokaraju, Philip Gold, Raphael Goldacre, Arthur Goldsmith, Portia Goldsmith, Darren Gomersall, Lucia Gomez, Raquel Gomez-Marcos, Ali Gondal, Celia Gonzalez, Jack Goodall, Bob Goodenough, Laura Goodfellow, James Goodlife, Camelia Goodwin, Elizabeth Goodwin, Jayne Goodwin, Paula Goodyear, Rajiv Gooentilleke, Sharif Goolam-Hossen, Michelle Goonasekara, Sheila Gooseman, Shameer Gopal, Peter Gordon, Sally Gordon, Hugh Gorick, Caitlin Gorman, Claire Gorman, Stuart Gormely, Diana Gorog, Jan Gorry, Michelle Gorst, Thomas Gorsuch, Jayshreebahen Gosai, Rebecca Gosling, Sally Gosling, Georgina Gosney, Vanessa Goss, Dzintars Gotham, Naomi Gott, Elizabeth Goudie, Amanda Gould, Angela Gould, Susan Gould, Lysander Gourbault, Anna Gouveia, Abha Govind, Sharon Gowans, Girish Gowda, Rohit Gowda, Pauline Gowdy, Hannah Gower, Thomas Gower, Pankaj Goyal, Sunil Goyal, Sushant Goyal, Beverley Graham, Clive Graham, Jane Graham, Jonathan Graham, Justin Graham, Libby Graham, Sharon Graham, Matthew Graham-Brown, Julia Grahamslaw, Gianluca Grana, Tracyanne Grandison, Louis Grandjean, Alison Grant, Ann Grant, David Grant, Matthew Grant, Pauleen Grant, Rhys Gravell, Jenny Graves, Alasdair Gray, Catherine Gray, Georgina Gray, Harriet Gray, Ingrid Gray, Jackie Gray, Karen Gray, Nicola Gray, Sebastian Gray, Alan Grayson, Patricia Grealish, Fiona Greaves, Jack Greaves, Paul Greaves, Charlotte Green, Christopher Green, David Green, Frederick Green, Grace Green, Joel Green, Marie Green, Nicola Green, Stacey Green, Teresa Green, Diarra Greene, Philippa Greenfield, Alan Greenhalgh, Maraneka Greenslade, Daniel Greenwood, Sandra Greer, James Gregory, Jane Gregory, Katie Gregory, Tamsin Gregory, James Gregson, Jill Greig, Julia Greig, Rebecca Grenfell, Teena Grenier, Susan Grevatt, Glaxy Grey, Andrew Gribbin, Amy Gribble, Beverley Grice, Natasha Grieg, Douglas Grieve, Ben Griffin, Denise Griffin, Mel Griffin, Sian Griffith, Alexandra Griffiths, Andrew Griffiths, Daniel Griffiths, David Griffiths, Donna Griffiths, Isabel Griffiths, Mark Griffiths, Nicola Griffiths, Oliver Griffiths, Sarah Griffiths, Sharon Griffiths, Yvonne Griffiths, Sofia Grigoriadou, Steph Grigsby, Paul Grist, Stephen Grist, Evelina Grobovaite, Clarissa Grondin, Rachel Groome, Liliana Grosu, Jenny Grounds, Margaret Grout, Helen Grover, Jayne Groves, Neil Grubb, Julie Grundy, Francesca Guarino, Sharada Gudur, Sharazeq Guettari, Shivang Gulati, Vikas Gulia, Pumali Gunasekera, Malin Gunawardena, Kirun Gunganah, Jessica Gunn, Emma Gunter, Alok Gupta, Atul Gupta, Rajeev Gupta, Richa Gupta, Rishi Gupta, Tarun Gupta, Vineet Gupta, Ankur Gupta-Wright, Victoria Guratsky, Alvyda Gureviciute, Sambasivarao Gurram, Bhawana Gurung, Shraddha Gurung, Hazel Guth, Ruth Habibi, Berkin Hack, Pamela Hackney, Christian Hacon, Aiman Haddad, Denise Hadfield, Michalis Hadjiandreou, Nikolaos Hadjisavvas, Leah Hadzik, Anna Haestier, Nauman Hafiz, Rana Hafiz-Ur-Rehman, Javed Hafsa, Samantha Hagan, Jack W Hague, Rosemary Hague, Kate Haigh, Christina Haines, Scott Hainey, Morton Hair, Brigid Hairsine, Juraj Hajnik, Anne Haldeos, Writaja Halder, Jennie Hale, Carmel Halevy, Paul Halford, William Halford, Alistair Hall, Anthony Hall, Claire Hall, Elizabeth Hall, Emma Hall, Fiona Hall, Helen Hall, Jennifer Hall, Kathryn Hall, Bill Hall, Jan Hallas, Kyle Hallas, Charles Hallett, Becky-Lee Halls, Heather Halls, Maryam Hamdollah-Zadeh, Bilal Hameed, Imran Hamid, Mohamad Hamie, Bethany Hamilton, Fergus Hamilton, Leigh Hamilton, Nicola Hamilton, Ruth Hamlin, Eleanor Hamlyn, Beatrice Hammans, Shirley Hammersley, Kate Hammerton, Bev Hammond, Leah Hammond, Sara Hammond, Fiona Hammonds, Ibrahim Hamoodi, Karen Hampshire, Elizabeth Hampson, Jude Hampson, Lucy Hampson, Ozan Hanci, Ian Hancock, Sadiyah Hand, Jasmine Handford, Soran Handrean, Sarah Haney, Sheharyar Hanif, E Hanison, Alison Hanlon, Jennifer Hannah, Amy Hannington, Merhej Hannun, Aidan Hanrath, Anita Hanson, Jane Hanson, Kathryn Hanson, Steve Hanson, Helen Hanwell, Mazhar Ul Haq, Ala Haqiqi, Monjurul Haque, Lesley Harden, Zoe Harding, Simon Hardman, Joanna Hardy, Kumar Haresh, Rachel Harford, Beverley Hargadon, Carolyn Hargreaves, Emily Hargreaves, James Hargreaves, Alice Harin, Mohammed Haris, Edward Harlock, Sandra Harlow, Paula Harman, Tracy Harman, Mark Harmer, Muhammad A Haroon, Charlie Harper, Fiona Harper, Heather Harper, Melanie Harper, Peter Harper, Rosemary Harper, Sarah Harrhy, Sian Harrington, Yasmin Harrington-Davies, Jade Harris, Jess Harris, John Harris, Laura Harris, Marie-Clare Harris, Naomi Harris, Nichola Harris, Sophie Harris, Alex Harrison, David Harrison, Julie Harrison, Laura Harrison, Melanie Harrison, Rowan Harrison, Susie Harrison, Thomas Harrison, Wendy Harrison, Elizabeth Harrod, Ciaran Hart, Dominic Hart, Lisa Hartley, Rosemary Hartley, Ruth Hartley, Tom Hartley, William Hartrey, Phillipa Hartridge, Stuart Hartshorn, Heli Harvala, Alice Harvey, Angela Harvey, Max Harvey, Catherine Harwood, Helen Harwood, Brigitte Haselden, Kazi Hashem, Mohammed Hashimm, Tadaaki Hashimoto, Imranullah Hashmi, Sarah Haskins, Zena Haslam, Adil Hassan, Ali Hassan, Wagae UI Hassan, Waqar Ul Hassan, Sapna Hassasing, Jane Hassell, Philip Hassell, Alex Hastings, Bethany Hastings, Janice Hastings, Stephanie Hatch, Jonathan Hatton, Sheryl Haviland, May Havinden-Williams, Stefan Havlik, Daniel B Hawcutt, Kadean Hawes, Liz Hawes, Nicola Hawes, Annie Hawkins, Catherine Hawkins, Nancy Hawkins, Tanya Hawkins, Dan Hawley, Ed Hawley-Jones, Edward Haworth, Cathy Hay, Amna Hayat, Jamal Hayat, Mohamed-Riyal Hayathu, Tamsin Haydon, Anne Hayes, Jonas Hayes, Kate Hayes, Melony Hayes, Vanessa Hayes, Fiona Hayes, Patrick Hayle, Chloe Haylett, Antara Hayman, Melanie Hayman, Matthew Haynes, Richard Haynes, Rachel Hayre, Sarah Haysom, James Hayward, Patrick Haywood, Tracy Hazelton, Phoebe Hazenberg, Zhengmai He, Elizabeth Headon, Carrie Heal, Brendan Healy, Amy Hearn, Angela Heath, Rowan Heath, Diane Heaton, Kerry Hebbron, Gemma Hector, Andy Hedges, Katrine Hedges, Cheryl Heeley, Elaine Heeney, Rajdeep Heire, Ulla Hemmila, Cassie Hemmings, Scott Hemphill, Deborah Hemsley, Abigail Henderson, Jennifer Henderson, Steven Henderson, Lee Hennen, Kathryn Hennessy, Natalie Hennesy, Carol Ann Henry, Joanne Henry, Karol Henry, Lavinia Henry, Margo Henry, Natalie Henry, David Henshall, Mike Herbert, Gillian Herdman, Rosaleen Herdman-Grant, Morag Herkes, Emma Heron, Kay Heron, William Herrington, Emilia Heselden, Peta Heslop, Sharnie Beth Hesson, Simon Hester, Emily Hetherington, Joseph Hetherington, Chamila Hettiarachchi, Pramodh Hettiarachchi, Hayley Hewer, John Hewertson, Anna Hewetson, Sue Hewins, Jacqueline Hewitson, Claire Hewitt, Davina Hewitt, Richard Hewitt, Robert Heyderman, Nicolette Heydon, Mathis Heydtmann, Joseph Heys, Jonathan Heywood, Gareth Heywood-Beldon, Meg Hibbert, John Hickey, Naomi Hickey, Peter Hickey, Alex Hicks, Jenny Hicks, Rosie Hicks, Scott Rory Hicks, Daniel Higbee, Lucy Higgins, Andrew Higham, Martin Highcock, Judith Highgate, Mondy Hikmat, Alison Hill, Amanda Hill, Helen Hill, Joanne Hill, Lisa Hill, Martin Hill, Phoebe Hill, Uta Hill, Annette Hilldrith, Elizabeth Hillerby, Catherine Hillman-Cooper, Elisabeth Hilton, Zoe Hilton, Sarah Hinch, Marcus Hinde, Andrew Hindle, Alice Hindmarsh, Paul Hine, Kim Hinshaw, Clare Hird, Alison Hirst, Jemma Hives, Benson Ho, Michaela Hoare, David Hobden, Gill Hobden, Maria Hobrok, Simon Hobson, Renate Hodge, Simon Hodge, Lesley Hodgen, Holly Hodgkins, Louise Hodgkinson, Sally Hodgkinson, David Hodgson, Helen Hodgson, Luke Hodgson, Sheila Hodgson, Gemma Hodkinson, Kenneth Hodson, Matthew Hogben, Lucy Hogg, Lee Hoggett, Abigail Holborow, Catherine Holbrook, Catherine Holden, Melinda Holden, Thomas Holder, Niels Holdhof, Hannah Holdsworth, Lisa Holland, Maureen Holland, Nicky Holland, Marie Hollands, Elizabeth Holliday, Nina Holling, Gillian Hollis, Laszlo Hollos, Linda Holloway, Simon Holloway, Marcus Hollyer, Amy Holman, Ann Holmes, Benjamin Holmes, Megan Holmes, Raphael Holmes, Rebecca Holmes, Kelly Holroyd, Caroline Holt, Lyndsey Holt, Siobhan Holt, Susie Holt, Alexandra Holyome, Marie Home, Toni Home, Renate Homewood, Kate Hong, Laura Hontoria del Hoyo, Clare Hooper, Sarah Hoosdally, Samantha Hope, Susan Hope, Bridget Hopkins, Peter W Horby, Stephanie Horler, Anil Hormis, Daniel Hornan, Nicola Hornby, Zoey Horne, Latoya Horsford, Megan Horsford, Mark Horsford, Valana Horsham, Alexander Horsley, Ashley Horsley, Elizabeth Horsley, Sarah Horton, Nicola Horton-Turner, Jane Hosea, Toby Hoskins, Muhammad S Hossain, Rashed Hossain, Leanne Hostler, Maxine Hough, Sarah Hough, Brittany Houghton, Catherine Houghton, Iain Houghton, Kathryn Houghton, Rebecca Houlihan, Angela Houston, Hamish Houston, Tawedzegwa Hove, Roseanna Hovvels, Lee How, Laura Howaniec, Laura Howard, Linda Howard, Lucy Howard, Sarah Howard, Stuart Howard, Richard Howard-Griffin, Alison Howarth, Diane Howarth, Serena Howe, Mark Howells, Lyn Howie, Kerry Howlett, Sophie Howlett, Joanne Hoyle, Josh Hrycaiczuk, Naing Zaya Htoon, Su Htwe, Ying Hu, Chiang Ooi Huah Huah, Abby Huckle, Shahzya Huda, Alison Hudak, Lisa Hudig, Alex Hudson, Cara Hudson, Heather Hudson, Peter Hudson, Oli Hudson, Alison Hufton, Connor Huggins, Alistair Hughes, Eithne Hughes, Emma Hughes, Gareth Hughes, Heather Hughes, Luke Hughes, Rachel Hughes, Rebecca Hughes, Samantha Hughes, Stephen Hughes, Vikki Hughes, Wesley Hughes, Lukas Huhn, Ching Hui, Ruth Hulbert, Diana Hull, Grace Hull, Robert Hull, Amanda Hulme, Peter Hulme, Wendy Hulse, George Hulston, Ryan Hum, Laura Humber, Megan Hume, Charlotte Humphrey, Ismay Humphreys, Alasdair Humphries, Joanne Humphries, Lena Hunold, Fiona Hunt, Kristen Hunt, Luke Hunt, Sophie Hunt, Al Hunter, Alexandra Hunter, Isobel Hunter, Karl Hunter, Neil Hunter, George Huntington, Elizabeth Hurditch, Cian Hurley, Katrina Hurley, Mohammed A Husain, Syeda Yusra Husaini, Coralie Huson, Afreen Hussain, Ibraar Hussain, Ifza Hussain, Mohammad Hussain, Muhammad Hussain, Reda Hussain, Sajid Hussain, Samia Hussain, Sanniah Hussain, Wasim Hussain, Yasmin Hussain, Mohammed Hussam El-Din, Raheem Hussein, Rebecca Hussey, Camille Hutchinson, Dorothy Hutchinson, Elizabeth Hutchinson, John Hutchinson, Claire Hutsby, Paula Hutton, Daniella Hydes, Jamie Hyde-Wyatt, Niamh Hynes, Megan Hyslop, Mazen Ibraheim, Abdalla Ibrahim, Ahmed Ibrahim, Asil Ibrahim, Mohamed Ibrahim, Monzeer Ibrahim, Wadah Ibrahim, Adetokunbo I Idowu, Muhammad Idrees, Hina Iftikhar, Mawara Iftikhar, Chukwuemeka Igwe, Mohammad Ijaz, Amaju Ikomi, Clare Iles, Stamatina Iliodromiti, Mary Ilsley, Lorna Ilves, La'ali Imam-Gutierrez, Christopher Imray, Alison Imtiaz, Haider Imtiaz, Claire Ingall, Jack Ingham, Julie Ingham, Rory Ingham, Tejas Ingle, Jennifer Inglis, Anne Ingram, Luke Ingram, Peter Inns, Ken Inweregbu, Andreea A Ionescu, Ana Ionita, Ilian P Iordanov, Anil Ipe, Adil Iqbal, Madiha Iqbal, Mohammed Iqbal, Faisal Iqbal Sait, Jane Ireland, Robert Irons, Mohannad Irshad, Muhammad S Irshad, Janice Irvine, Val Irvine, Pamela Irving, Robert Irving, Mina Ishak, Erica Isherwood, Aminul Islam, Abdurrahman Islim, Ali Ismail, Omar Ismail, Caroline Ison, M'hamedi Israa, Sharon Isralls, Ali Issa, Monica Ivan, Catrina Ivel, Chineze Ivenso, Ashleigh Ivy, Sophie Iwanikiw, Karen Ixer, Menaka Iyer, Mia Iyer, Calum Jack, Amanda Jackson, Anthony Jackson, Ben Jackson, Beth Jackson, Douglas Jackson, Ella Jackson, Hayley Jackson, Helen Jackson, Jane Jackson, Julie Jackson, Karin Jackson, Lauren Jackson, Melanie Jackson, Nicola Jackson, Shane Jackson, Sharon Jackson, Nikita Jacob, Patricia Jacob, Reni Jacob, Nicola Jacques, Terri-Lisa Jacques-Brown, Anisa Jafar, Daniel Jafferji, Ali Jaffery, Chandrashekar Jagadish, Vijay Jagannathan, Sam Jaggard, Mandeep Jagpal, Fernandez R Jaime, Neemisha Jain, Seema Jain, Susan Jain, Sanjay Jaiswal, Danyal Jajbhay, Thomas Jaki, Bintou Jallow, Yusuf Jaly, Sabine Jamal, Zeba Jamal, Yasmin Jameel, Albie James, Christie James, Kate James, Lee James, Linda James, Mark James, Nicholas James, Olivia James, Rebecca James, Ruth James, Tracy James, Jack Jameson, Aaron Jamison, Phoebe Jane, Azara Janmohamed, Sabrina Jansz, Deepa Japp, Lorraine Jappy, Victor Jardim, Catherine Jardine, Emma Jarnell, Ellie Jarvie, Ann Jarvis, Claire Jarvis, Lisa Jarvis, Rosina Jarvis, Patrycja Jastrzebska, Hafsa Javed, Mays Jawad, Lona Jawaheer, Kauky Jawaid, Anu Jayachandran, Dinakaran Jayachandran, Angelina Jayakumar, Deepak Jayaram, Ravi Jayaram, Geeshath Jayasekera, Thilina Jayatilleke, Abi Jayebalan, Saman Jeddi, Vandana Jeebun, Mohammad S Jeelani, Zeynab Jeewa, Emma Jefferson, Katie Jeffery, Helen Jeffrey, Jenni Jeffrey, Rachel Jeffrey, Sue Jeffrey, Nathan Jeffreys, Benjamin Jeffs, Debbie Jegede, Taylor Jemima, Ifan Jenkin, Alison Jenkins, Christopher Jenkins, David Jenkins, Elinor Jenkins, Sarah Jenkins, Sian Jenkins, Stephen Jenkins, Jacqui Jennings, Louise Jennings, Rebecca Jennings, Virginia Jennings, Ellen Jerome, Douglas Jerry, Ellen Jessup-Dunton, Jorge Antonio Jesus Silva, Champa Jetha, Kishan Jethwa, Jeby Jeyachandran, Visuvanathan Jeyakumar, Dharshana Jeyapalan, Shaman Jhanji, Khoo Jian, Zhixin Jiao, Laura Jimenez, Ana Jimenez Gil, Jithin Jith, Teishel Joefield, Navraj Johal, Karine Johannessen, Aisyah Johari, Annie John, Anu John, Navin John, Sarah John, Emma Johns, Margaret Johns, Anne-Marie Johnson, Antoinette Johnson, David Johnson, Emma Johnson, Gillian Johnson, Kathryn Johnson, Katie Johnson, Luke Johnson, Mark Johnson, Nelsonseelan Johnson, Oliver Johnson, Rachel Johnson, Tracy Johnson, Zoe Johnson, Claire Johnston, Janet Johnston, Laura Johnston, Susan Johnston, Victoria Johnston, Dawn Johnstone, Ed Johnstone, Janet Johnstone, Manohar Joishy, Adam Jones, Alistair Jones, Annabel Jones, Ben Jones, Bryony Jones, Carys Jones, Ceri Jones, Charlotte Jones, Christine E Jones, Debra Jones, Emily Jones, Gareth Jones, Geraldine Jones, Hazel Jones, Jac Jones, James Jones, Jamie Jones, Jessica Jones, Jonathon Jones, Julie Jones, Karen Jones, Kate E Jones, Kevin Jones, Laura Jones, Laura M Jones, Lorna Jones, Louise Jones, Mathew Jones, Nicola Jones, Paul Jones, Rhianna Jones, Ruth E Jones, Samantha Jones, Sophie Jones, Stefanie Jones, Steve Jones, Taya Jones, Tim Jones, Tracey Jones, Ramya Jonnalagadda, Rebecca Jordache, Annette Jose, Sanal Jose, Anna Joseph, Joseph Joseph, Rosane Joseph, Sibet Joseph, Dhaara Joshi, Mehul Joshi, Pratichi Joshi, Revati Joshi, Benz Josiah, Tiffany Joyce, Adriel Ju Wen Kwek, Edward Jude, Parminder Judge, Jessica Juhl, Sirisha Jujjavarapu, Mark Juniper, Edmund Juszczak, Deepthi Jyothish, Kasamu Kabiru Dawa, Mark Kacar, Katarina Kacinova, Nikhil Kadam, Rebecca Kahari, Gail Kakoullis, Azad Kala Bhushan, Richard JK Kalayi, Roobala Kaliannan Periyasami, Efthymia Kallistrou, Seika Kalsoom, Elisa Kam, John Kamara, Mohamed Kamara, Ajay Kamath, Prakash Kamath, Ravindra Kamath, Siddharth Arun Kamerkar, Nick Kametas, Musaiwale Kamfose, Arul Kandaswamy, Leia Kane, Osei Kankam, Thogulava Kannan, Abhinav Kant, Vikas Kapil, Ritoo Kapoor, Sonal Kapoor, Sourjya Kar, Janaka Kara, Vasita Kara, Marina Karakantza, Rona Kark, Nicholas Karunaratne, Natashja Kasianczuk, Vidya Kasipandian, Rizwan Kassam, Janarth Kathirgamachelvam, Victoria Katsande, Kulbinder Kaul, Daljit Kaur, Dervinder Kaur, Jasmin Kaur, Jaspreet Kaur, Satvinder Kaur, Zunaira Kausar, Mohammad AA Kawser, Andrea Kay, Sarah Kay, Jossy N Kayappurathu, Callum Kaye, Ahemd Kazeem, Naved Kazi, Sharon Kaznica, Samantha Kearley, Rachel Kearns, Nichola Kearsley, Joanne Keating, John Keating, Liza Keating, Elizabeth Keddie-Gray, Katie Keen, Natalie Keenan, Jonathan Kefas, Stephen Kegg, Laura Keith, Uzoamaka Keke, Tosin Kelani, Joanne Kellett, Jeremy Kellington, Alison Kelly, Conor Kelly, David Kelly, Diane Kelly, Dominic Kelly, Emma Kelly, Laura Kelly, Martin Kelly, Michael Kelly, Rosalind Kelly, Sinead Kelly, Stephen Kelly, Thomas Kelly, Mary Kelly-Baxter, Marketa Keltos, Timothy Kemp, Kelly Kemsley, Alexandra Kendall-Smith, Sarah Kennard, Ann Kennedy, Caroline Kennedy, James Kennedy, Sophie Kennedy-Hay, Julia Kenny, Kelly Kent, Melanie Kent, Lynne Keogan, Alexander Keough, Clement Kerlin, A Kerr, Andrew Kerr, Maria Kerr, Caroline Kerrison, Anthony Kerry, Samantha Kershaw, Helen Kerslake, Ian Kerslake, Helen Kerss, Jocelyn Keshet-Price, Margaret Kevern, Georgina Keyte, Abdul Khadar, Ali Khalid, Muhammad U Khalid, Syed Khalid, Amir Khalil, Asma Khalil, Sijjad Khalil, Abubakar Khan, Ali Khan, Al-Imran Khan, Arham Khan, Asad Khan, Aurangzeb Khan, Burhan Khan, Camran Khan, Fatimah Khan, Kausik Khan, Malik Aamaz Khan, Marria Khan, Mehrunnisha Khan, Mohammad Khan, Mohammed Khan, Nayeem Khan, Omar Khan, Rahe Khan, Rahila Khan, Sabiya Khan, Shabana Khan, Shahul Khan, Shoaib Khan, Tasaduksultan Khan, Waseem Khan, Usman F Khatana, Jibran Khatri, Jyoti Khatri, Hafiza Khatun, Taslima Khatun, Mena Kheia, Jacyntha Khera, Htet Ei Khin, Najaf Khoja, Kiran Khokhar, Jayne Khorsandi, Chloe Khurana, Faith Kibutu, Andrew Kidd, Michelle Kidd, Joe Kidney, Shane Kidney, Will Kieffer, James Kilbane, Caroline Kilby, Eileen Killen, Susan Kilroy, Bomee Kim, Jee Whang Kim, Sarah Kimber, Andy King, Barbara King, Jennifer King, Kirsten King, Rachel King, Sarah King, Tony King, Victoria King, Emily King-Oakley, Laura Kingsmore, Andy King-Venables, Fiona Kinney, Sidra Kiran, Jeremy Kirk, Jodie Kirk, Daniel Kirkbride, Amy Kirkby, Ian Kirker, Emily Kirkham, Gemma Kirkman, Ursula Kirwan, Kelly Kislingbury, Toby Kitching, Laura Kitto, Lauren Kittridge, Sarah Klaczek, Frieder Kleemann, Susan Kmachia, Chris Knapp, Lucy Knibbs, Alicia Knight, Fraser Knight, Marian Knight, Sarah Knight, Steven Knight, Tom Knight, Ellen Knights, Jane Knights, Toby Knights, Martin Knolle, Carol Knott, Charlotte Knowles, Karen Knowles, Laurence Knowles, Emily Knox, Lucy Knox, Oliver Koch, Ronan Kodituwakku, Gouri Koduri, Aisha Koirata, Eirene Kolakaluri, Magdalena Kolodziej, Eirini Kolokouri, Keith Kolsteren, Samantha Kon, Niladri Konar, Mari Kononen, Athanasios Konstantinidis, Hui Fen Koo, Imogen Koopmans, Emmanuela Kopyj, Laura Korcierz, James Korolewicz, George Koshy, Chris Kosmidis, Jalpa Kotecha, Easwari Kothandaraman, Leonidas Koukouflis, Koushan Kouranloo, Rukhsana Kousar'c, Margarita Kousteni, Maja Kovac, Alex Kozak Eskenazia, Kestutis Krasauskas, Raghu Krishnamurthy, Vinodh Krishnamurthy, Manju Krishnan, Hari Krishnan, Suzanne Krizak, Sean Krupej, Agnieszka Kubisz-Pudelko, Soren Kudsk-Iversen, Aurimas Kudzinskas, Chirag Kukadiya, Nainesha Kulkarni, Aditi Kumar, Mayur Kumar, Ramesh Kumar, Ravi Kumar, Rita Kumar, Rupa Kumar, Satish Kumar, Vimal Kumar, Arun Kundu, Heinke Kunst, Amit Kurani, Mohammed Kurdy, Rincy Kurian, Vimal Kurmars, Cameron Kuronen-Stewart, Ranganai S Kusangaya, Vlad Kushakovsky, Mandy Kuunal, Apexa Kuverji, Amma Kyei-Mensah, Thyra Kyere-Diabour, Moe Kyi, Nyan M Kyi, Laura Kyle, Laura Kyle, Karali-Tsilimpari Kyriaki, Julius Labao, Louise Lacey, Nikki Lack, Emma Ladlow, Heather Lafferty, Shondipon Laha, Sushil Lahane, Clement Lai, James Lai, Emma Laing, Robert Laing, Inez Laing-Faiers, Emily Laity, Michelle Lake, Nicki Lakeman, David Lalloo, Fiona Lalloo, Alison Lam, Fiona Lamb, Lucy Lamb, Thomas Lamb, Nick Lambe, Pauline Lambert, Claudia Lameirinhas, Mohammed KG Lami, Abigail Lamikanra, Holly Lamont, Michal Lamparski, Djillali Lamrani, Christine Lanaghan, Rebecca Lanaway, Ivone Lancona-Malcolm, Julia Lancut, Geraldine Landers, Martin J Landray, Matthew Lane, Nicholas Lane, Alidih Lang, Stephen Lang, Daniel Langer, Margaret Langley, Charles Langoya, Emily Langthorne, Taiya Large, Wojciech Lason, Anna Last, Scott Latham, John Latham-Mollart, Afzal Latheef, Darren Latimer, Nang Latt, Carly-Jane Lattimore, Dawn Lau, Eva Lau, Myra Laurenson, Hou Law, Jennifer Law, Jessica Law, Penny Law, Richard Law, Colin Lawler, Mark Lawley, Emma Lawrence, Jo Lawrence, Neil Lawrence, Ryan Lawrie, Jemima Lawson, Joanne Lawson, Louise Lawson, Rebecca Lawton, Michael Lay, Christine Laycock, Reina Layug, Maria Lazo, Vietland Le, Amelia Lea, William Lea, Ian Leadbitter, Thomas Leahy, Richard Lean, Lorna Leandro, Darren Leaning, Sandra Leason, Christina Leaver, Marie Anne Ledingham, Emma Lee, Hannah Lee, Irish Lee, Judith Lee, Sam Lee, Shi Han Lee, Simon Lee, Sindy Lee, Stephanie Lee, Tracey Lee, Xiang Lee, Diana Lees, Jennifer Lees, Helen Legge, Julian Leggett, Katie Leigh-Ellis, Kevan Leighton, Nicky Leitch, Eleni Lekoudis, Petula Lemessy, Nicholas Lemoine, Joana Lemos, Irina Lenchuk, Katy Leng, Katrina Lennon, Liz Lennon, Isabel Lentell, Kelly Leonard, Wen Leong, Nicky Leopold, Oskar Lepiarczyk, Isla Leslie, Eleni Lester, Joe Leung, Ullrich Leuschner, Emma Levell, Chris Levett, Alice Lewin, Michaela Lewin, Alison Lewis, David Lewis, Dee Lewis, Georgina Lewis, Gillian Lewis, Joanne Lewis, Joseph Lewis, Kathryn Lewis, Keir Lewis, Leon Lewis, Lisa Lewis, Marissa Lewis, Rob Lewis, Robert Lewis, Catherine Lewis-Clarke, Lorraine Lewis-Prosser, Katherine Lewiston, Adam Lewszuk, Penny Lewthwaite, Samantha Ley, Anna Li, Jenny Li, Angela Liao, Victoria Licence, David Lieberman, Susan Liebeschuetz, Nicky Lightfoot, Patrick Lillie, Ben Lim, Carys Lim, Ee Thong Lim, Ivy Lim, Terence Lim, Wei Shen Lim, Wilson Lim, James Limb, Usha Limbu, Christian Linares, Dermot Linden, Gabriella Lindergard, Kate Lindley, Charlotte Lindsay, Emily Lindsay, Max Lindsay, Helen Lindsay-Clarke, Mirella Ling, Claire Lingam, Linette Linkson, Mike Linney, Louise Linsell, Conrad Lippold, George Lipscomb, Karen Lipscomb, Laura Lipskis, Ana Lisboa, Evangeline Lister, Charlotte Little, Jeff Little, Sam Little, Xuedi Liu, Alexandra Liversidge, Jill Livingstone, Daniel K Llanera, Rhiannon Llewellyn, Martin Llewelyn, Adam Lloyd, Aimee Lloyd, Arwel Lloyd, Oliver Lloyd, Richard Lloyd, Su Lo, David Loader, Cristina Lobato, Maria Lobo-Clarke, Lydianne Lock, Sara Lock, Stephen Lock, Angela Locke, Jacqueline Locke, Thomas Locke, Teresa Lockett, Sandy Lockyer, Jeorghino Lodge, Terrence Lodge, Martina Lofthouse, Tracey Lofting, Heather Loftus, Meg Logan, Chloe Logue, Sook Yin Loh, Siddharth Lokanathan, Kaatje Lomme, Emily London, Gabriella Long, Natalie Long, Bev Longhurst, Mark Longshaw, Jennifer Lonnen, Caroline Lonsdale, Laura Looby, Ronda Loosley, Paola Lopez, Paula Lopez, Robert Lord, Stuart Lord, Laura Lordache, Kieron Loregnard, Catherine Lorenzen, Claire Lorimer, Francesco Loro, Rachel Lorusso, Eva Loutraris, Robert Loveless, Maxine Lovell, Angeliki Loverdou, Huw Lovett, Daniel Loveys, Andrew Low, Jen Mae Low, Alastair Lowe, Caroline Lowe, Catherine Lowe, Emily Lowe, Faye Lowe, Michael Lowe, Linda Lowrey, Richard Lowsby, Vicki Lowthorpe, Emma Loxley, Gamu Lubimbi, Alexandra Lubina Solomon, Georgia Lucas, Jacob Lucas, Alice Lucey, Olivia Lucey, Suzanne Luck, H Luke, Jane Luke, Hayley Lund, Apurva Lunia, Muriel Lunn, Ji Luo, Julia Lussier, Cindy Nisha Luximon, Elisa Ly, Barrie Lyell, Elisavet Lyka, Sarah Lymn, Audrey Lynas, Ceri Lynch, Daniel Lynch, Daniella Lynch, Stephen Lynch, Helen Lyon, Rea-Grace Maamari, Hannah Mabb, Louies Mabelin, Jessica Macaro, Angela Macauley, Kateryna Macconaill, Chloe Macdonald, Stuart MacDonald, Tania MacDonald, Claire Macfadyen, James G Macfarlane, Jill Macfarlane, Laura Macfarlane, Cara MacGuigan, Lisa MacInnes, Iain MacIntyre, Jill MacIntyre, Kirsten Mack, Callum Mackay, Euan Mackay, Laura Mackay, Alexander Mackenzie, Matt Mackenzie, Robert MacKenzie Ross, Ami Mackey, Patricia Mackey, Fiona Mackie, Robert Mackie, Carolyn Mackinlay, Claire Mackintosh, Katherine Mackintosh, Sheila MacLennan, Mary Joan MacLeod, Michael Macmahon, Andrew MacNair, Catherine Macphee, Iain Macpherson, Catriona Macrae, Allan MacRaild, Eilidh MacVean, Alannah Madden, Mary Madden, Norman Madeja, Karen Madgwick, Pradeep Madhivathanan, Madhavi Madhusudhana, Harriet Madiyiko, Alpha Madu, Lorraine Madziva, Marion Mafham, Nick Magee, Frederick Magezi, Tim Maggs, Negar Maghsoodi, Christopher Magier, Marios Magriplis, Kathryn Maguire, Natasha Mahabir, Subramanian Mahadevan-Bava, Anjanie Maharajh, Ajit Mahaveer, Bal Mahay, Kanta Mahay, Hibo Mahdi, Thushika Mahendiran, Siva Mahendran, Sarah Maher, Anistta Maheswaran, Shameera Maheswaran, Tina Maheswaran, Parisa Mahjoob-Afag, Ahmed Mahmood, Farhana Mahmood, Waheed Mahmood, Zahra Mahmood, Hager Mahmoud, Ewan Mahony, Luke Mair, Toluwani Majekdunmi, Kesson Majid, Rupert Major, Jaydip Majumdar, Mohammad KH Majumder, Stephen Makin, Marius Malanca, Hannah Malcolm, Flora Malein, Neeraj Malhan, Ayesha Malik, Gulshan Malik, Mohammed Maljk, Paul Mallett, Petrina Mallinder, Georgia Mallison, Louise Mallon, Edward Malone, Gracie Maloney, Edgar Malundas, Madhu Mamman, Irene Man, Kathy Man, Rossana Mancinelli, Marco Mancuso-Marcello, Tracy Manders, Lauren Manderson, Justin Mandeville, Roope Manhas, Carmen Maniero, Ravi Manikonda, Bobby Mann, Jonathan Manning, Lynne Mannion, Katherine Mansi, Katarina Manso, Dina Mansour, Isheunesu T Mapfunde, Predeesh Mappa, Hemant Maraj, Garikayi Marange, Lisa March, Clare Marchand, Neil Marcus, Maria Marecka, Maria Marecka, Gomathi Margabanthu, Jordi Margalef, Lavinia Margarit, Georgios Margaritopoulos, Mike Margarson, Fernandez M Maria del Rocio, Teresa Maria Pfyl, Victor Mariano, Helen Maria-Osborn, Ashleigh Maric, Grace Markham, John Markham, Maria Marks, Pamela Marks, Elisabeth Marouzet, Arran Marriott, Cheryl Marriott, Nemonie Marriott, Brian Marsden, Karen Marsden, Paul Marsden, Sarah Marsden, Tracy Marsden, Robyn Marsh, Adam Marshall, Andrew Marshall, Gail Marshall, Henry Marshall, Jaimie Marshall, James Marshall, Jenna Marshall, Nicola Marshall, Riley Marshall, Jennifer Marshall, Samantha Marston, Emmeline Martin, Hayley Martin, Hope Martin, Jane Martin, Karen Martin, Kate Martin, Laila Martin, Michael Martin, Noelia Martin, Tim Martin, Winston Martin, Sarah Martin, Tim Martindale, Marcus Martineau, Alexander Martinez, Lauren Martinez, Jose Carlos Martinez Garrido, Juan Martin-Lazaro, Olivia Martins, Lucas Martins Ferreira, Vijay Kumar Maruthamuthu, Gemma Maryan, Roman Mary-Genetu, Sam Maryosh, Vidan Masani, Diego Maseda, Sheila Mashate, Yasaman Mashhoudi, Al Mashta, Izhaq Masih, Sanna Masih, Nick Maskell, Perry Maskell, Matthew Masoli, Lynn Mason, Rebecca Mason, Richard Mason, Ruth Mason, Claire Mason, Mohammad Masood, Mohammad T Masood, Syed Masood, Syed SME Masood, Aaqib Masud, Lear Matapure, Cristina Matei, Ropafadzo Matewe, Manraj Matharu, Stephy Mathen, Alex Mather, Nicole Mather, Jonathan Mathers, Joanna Matheson, Amal Mathew, Anna Mathew, Moncy Mathew, Verghese Mathew, Caroline Mathews, Jesha Mathews, Kate Mathias, Marion Mathie, Darwin Matila, Wadzanai Matimba-Mupaya, Nashaba Matin, Elina Matisa, Max Matonhodze, Elijah Matovu, Jaysankar Mattappillil, Alison J Matthews, Heather Matthews, Helen Matthews, Sue Matthews, Gwynn Matthias, Fiona Maxton, Adam Maxwell, Gemma Maxwell, Veronica Maxwell, James May, Joanne May, Oliver May, Philippa May, Irving Mayanagao, Matthew Maycock, Graham Mayers, Lee Maynard, Shelley Mayor, Ibreaheim Mazen, Andrea Mazzella, Nyambura Mburu, Mercy Mbwembwe, Martyn McAdam, Eleanor McAleese, Helinor McAleese, Paul McAlinden, Audrey McAlpine, Graeme McAlpine, Jonathan McAndrew, Hamish McAuley, Sarah McAuliffe, Claire McBrearty, Carole McBride, Erin McBride, Michael McBuigan, James McBurney, Laura McCabe, Amanda McCairn, Martina McCalmont, Jake McCammon, Nicole McCammon, Conor McCann, Alexandra McCarrick, Brendan McCarron, Eoghan McCarthy, Michelle McCarthy, Natalie McCarthy, Sinead McCaughey, Gareth McChlery, Tara McClay, Beverley McClelland, Declan McClintock, Patricia McCormack, Jacqueline McCormick, Wendy McCormick, Paul McCourt, Jame McCrae, Sharon McCready, Allison McCreath, Gordan McCreath, Helen McCreedy, Louise McCreery, Iain J McCullagh, Josephine McCullagh, Liz McCullagh, Megan McCullagh, Conor McCullough, Katherine McCullough, Nicola McCullough, Sarah McCullough, Fiona McCurrach, Rory McDermott, Katharine McDevitt, Helen McDill, Basil McDonald, Claire McDonald, Debbie McDonald, Rob McDonald, Sam McDonald, Damhnaic McDonald, Rowan McDougall, Irene McEleavy, Julie McEnerney, Julie McEntee, Evanna McEvoy, Ruth McEwen, Margaret McFadden, Denise McFarland, Margaret McFarland, Rachel McFarland, Erin McGarry, Lorcan McGarvey, Margaret McGarvey, Clodagh McGettigan, Michael McGettrick, Christopher McGhee, Fiona McGill, Sarah McGinnity, Hannah McGivern, Neil McGlinchey, Phil McGlone, Deborah McGlynn, Claire McGoldrick, Clare McGoldrick, Elizabeth McGough, Margaret McGovern, Brendan McGrath, Amanda McGregor, Annemarie McGregor, Cathryn McGuinness, Heather McGuinness, Sean McGuire, Tara McHugh, Caroline McInnes, Neil McInnes, Karen McIntyre, Mhairi McIntyre, Carolyn McKay, Lorna McKay, Conor P McKeag, Madeleine McKee, Joseph McKeever, Shirley McKenna, Donogh McKeogh, Denise Mckeown, Caroline McKerr, Anthony M McKie, Claire Mckie, Laura Mckie, Gerard McKnight, Heather McLachlan, Andrew McLaren, Barbara McLaren, Nicola McLarty, Maria McLaughlin, James McLay, Mary McLeish, Tina McLennan, Lorna McLintock, Stewart McLure, Amanda McMahon, Anne Marie McMahon, Genevieve McMahon, Joanne McMahon, Mike McMahon, Stephen McMahon, Terence McManus, Moyra McMaster, Paddy McMaster, Samuel McMeekin, Nicola McMillan, Jason McMinn, Liam McMorrow, Helen McNally, Darren McNamara, Helen Mcnamara, Deborah McNaughton, Fiona McNeela, Lynne McNeil, Claire McNeill, Shea McNeill, Una McNelis, Mary P McNicholl, Melanie McNulty, Roisin McNulty, Christopher McParland, Mark McPhail, Alison McQueen, Anna McSkeane, Denise McSorland, Gini McTaggart, Jacqueline McTaggart, Sam McWilliam, Joanna Mead, Karen Mead, Emma Meadows, Olivia Meakin, Ben Mearns, Claire Mearns, Kim Mears, William Mears, Manjula Meda, Ayren Mediana, Ross Medine, Thomas Medveczky, Sharon Meehan, Matthew Meek, Emily Meeks, Anke Meess, Abbi Megan, Nevan Meghani, Salim Meghjee, Jenny Mehew, Rohan Mehra, Jana Meier, James Meiring, Rayane Mejri, Sabina Melander, Adriana-Stefania Melinte, Stephanie Mellin, Francesca Mellor, Phil Mellor, Samantha Mellor, Zoe Mellor, Katrina Mellows, Vladimir Melnic, Alice Melville, Julie Melville, Helen Membrey, Mark Mencias, Cheryl Mendonca, Alexander Mentzer, Dan Menzies, Sue Mepham, Oliver Mercer, Pauline Mercer, Arwa Merchant, Fatema Merchant, Mihaela Mercioniu, Megan Meredith, Marta Merida Morillas, Blair Merrick, Jack Merritt, Simon Merritt, Ekta Merwaha, Simon Message, Gabriel Metcalf-Cuenca, Benjamin Metcalfe, Kneale Metcalfe, Stella Metherell, Greg Methven, Alexsandra Metryka, Louise Mew, Simon Meyrick, Nhlanhla Mguni, Atiqa Miah, Jagrul Miah, Nahima Miah, Gabriela Mic, Dariush Micallef, Alice Michael, Angiy Michael, Shery Michael, Vincent Michael, Natalia Michalak, Loredana Michalca-Mason, Claire Michelson, Janet Middle, Hayley Middleton, Jennifer T Middleton, Maeve Middleton, Sophie Middleton, Shelley Mieres, Gail Miflin, Loredana Mihalca-Mason, Theresia Mikolasch, Sarah Milgate, Colin Millar, Ian Millar, Jonathan Millar, David Miller, Johnathan Miller, Lucy Miller, Rachel Miller, Naomi Miller-Biot, Alex Miller-Fik, Louise Millett, Hazel Milligan, Iain Milligan, Caitlin Milliken, Katherine Millington, Samuel Millington, Melanie Milloy, Helen Mills, Janet Mills, Steve Mills, Helen Millward, Rebecca Miln, Alice Milne, Charlotte Milne, Louise Milne, Joanne Milner, Zayar Min, Samuel Mindel, Arron Minhas, Chrissie Minnis, Paul Minnis, Anne Minogue, Jane Minton, Frederico Miranda, Lucy Mires, Taimur Mirza, Anjum Misbahuddin, Aseem Mishra, Biswa Mishra, Eleanor Mishra, Ritu Mishra, Sannidhya Misra, Deena Mistry, Heena Mistry, Nita Mistry, Reena Mistry, Dushyant Mital, Sarah Mitchard, Alan Mitchell, Ben Mitchell, Piers Mitchell, Susan Mitchell, Philip Mitchelmore, Andrew Mitra, Atideb Mitra, Sandip Mitra, Clarisse Mizzi, Gloria Mmadubuko, Emma Moakes, Emma Moatt, Gita Modgil, Abdelrahman Mohamed, Arez Mohamed, Osab Mohamed, Waheed Mohammad, Aliabdulla Mohammed, Omer Mohammed, Yaser NS Mohammed, Bilal A Mohamud, Mahalakshmi Mohan, Amr Moharram, Rachel Moir, Jonathan Mok, Christine Moller-Christensen, Mateus Mollet, Malid Molloholli, Aoife Molloy, April Molloy, Linda Molloy, Andrew Molyneux, Tasnim Momoniat, Holly Monaghan, Josephine Monaghan, Krista Monaghan, Shiva Mongolu, Katelyn Monsell, Mahmoud Montasser, Alan Montgomery, Hugh Montgomery, Prebashan Moodley, Ian Moody, Margaret Moody, Nick Moody, Angela Moon, James Moon, Ji-Hye Moon, Maria Moon, May Moonan, Parvez Moondi, Alex Moore, Carly Moore, Christopher Moore, Davidjar Moore, Faye Moore, Gillian Moore, Hannah Moore, Judith Moore, Laura Moore, Sally Moore, Sonia Moore, Tracy Moore, Rachel Moores, Ed Morab, Jose Morales, Nuria Moramorell, Louise Moran, Grishma Moray, Jeronimo Moreno-Cuesta, Adam Morgan, Amy Morgan, Christine Morgan, Colin Morgan, Jackie Morgan, Lauren Morgan, Leila Morgan, Matthew Morgan, Patrick Morgan, Katie Morgan-Jones, Emily Morgan-Smith, Anna Morley, Thomas Morley, Wendy Morley, Anna Morris, Damian Morris, Fiona Morris, Helen Morris, Juliet Morris, Katie Morris, Laura Morris, Lucy Morris, Mary-Anne Morris, Niall Morris, Paul Morris, Sheila Morris, Susan Morris, Douglas Morrison, Moira Morrison, Mary Morrissey, Anna Morrow, Chantal Morrrell, Franca Morselli, Gordon Mortem, Chelsea Morton, Gordon Morton, Rosna Mortuza, Priti Morzaria, Alison Moss, Charlotte Moss, Rachel Moss, Sarah Moss, Stuart Moss, Nicki Motherwell, Johanna Mouland, Caroline Moulds, Hilary Moulton, Lorraine Mounsey, Elizabeth Mousley, Karen Moxham, Borja Moya, Quberkani Moyo, Eunice Mshengu, Sheila Mtuwa, Ali Muazzam, Iqtedar A Muazzam, Nykki Muchenje, Dalia Mudawi, Girish Muddegowda, Imran Mugal, Ahsan Mughal, Javaid Muglu, Javed Muhammad, Alison Muir, Carol Muir, Martin Muir, Dipak Mukherjee, Syed Asim Ali Mukhtar, Denise Mukimbiri, Tshinupay Mukwa, Peter Mulgrew, Ben Mulhearn, Arafat Mulla, Dee Mullan, Dileepkumar Mullasseril Kutten, Niall Mullen, Rosemary Mullett, Sandra Mulligan, Barbara Mullin, Joanne Mullings, Lana Mumelj, Andrew Mumford, Sarah Mumford, Mohammed Munavvar, Henry Munby, Anne-Marie Munro, Sheila Munt, Nafissah Munu, McDonald Mupudzi, Arshid Murad, Oluwatosin H Muraina, Koteshwara Muralidhara, Diane Murdoch, Mhairi Murdoch, Jennifer Murira, Alison Murphy, Carl Murphy, Emily Murphy, Fidelma Murphy, Gail Murphy, Jo Murphy, Peter Murphy, Sheenagh Murphy, Simon Murphy, Clare Murray, David Murray, Eleanor Murray, Katie Murray, Kenneth Murray, Lisa Murray, Lorna Murray, Tracey Murray, Eoin Murtagh, Mithun Murthy, Catherine Murton, Rosie Murton, Neeka Muru, Rosemary Musanhu, Maimuna Mushabe, Kaiser Mushtaq, Omaisa Mushtaq, Ahmed M Mustafa, Elhaytham Mustafa, Mustafa Mustafa, Ibrahim Mustapha, Zhain Mustufvi, Callum Mutch, Eric Mutema, Balakumar Muthukrishnan, Sheree Mutton, Natasha Muzengi, Memory Mwadeyi, Bettina Mwale, Esther Mwaura, Raji Myagerimath, Alice Myers, Sam Myers, Khin Swe Myint, Yadee Myint, Libor Myslivecek, Helen Nabakka, Evelyn Nadar, Iftikhar Nadeem, Moosa Nadheem, Asma Naeem, Hassan Naeem, Salman Naeem, Samraiz Nafees, Mohamed Nafei, Parminder Naga, Thapas Nagarajan, Imrun Nagra, Deepak Nagra, Mina Naguib, Kirushthiga Naguleswaran, K Shonit Nagumantry, Kevin Naicker, Sarveshni Naidoo, Gireesha Naik, Rishi Naik, Samir Naik, Devu S Nair, Rajiv Nair, Tanushree Nair, Jay Naisbitt, Kerry Naismith, Sri Nallapareddy, Soum Nallapeta, Arumugan Nallasivan, Uttam Nanda, Aarti Nandani, Ali Raza Naqvi, Asadullah Naqvi, Sara Naqvi, Shruthi Narayan, Sophia Nasa, Dominic Nash, Nader Nasheed, Abdul Nasimudeen, Umer Nasir, Marwan Nassari, Tahir Nasser, Anushka Natajaran, Anuja Natarajan, Geetha Natarajan, Nalin Natarajan, Nikhila Natarajan, Rajkumar Natarajan, Noel Nathaniel, Mala Nathvani, Priyan Nathwani, George Nava, Neena Navaneetham, Jeya Navaratnam, Helen Navarra, Sadaf Naveed, John Navin, Khuteja Nawaz, Sarfaraz Nawaz, Shasta Nawaz, Bonilla Nayar, Suzanne Naylor, Moez Nayyar, Farrah Naz, Mobeena Naz, Salima Nazarali, Babak Nazari, S Nazir, Sehar Nazir, Dumisani Ncomanzi, Onyine Ndefo, Alan Neal, Elaine Neary, Mostafa Negmeldin, Paula Neill, Hector E Neils, Avideah Nejad, Louise Nel, Marie Nelson, Richard Nelson, Scott Nelson, Rajesh Nemane, Samiksha Nepal, Daniel Nethercott, Kimberley Netherton, Kimberley Nettleton, Claire-Michelle Neville, Tracy Nevin, Josephine Newanji, Alison Newby, Angela Newby, David Newby, Tracy Newcombe, Charlotte Newman, Diana Newman, Julie Newman, Oscar Newman, Richard Newman, Tabitha Newman, Thomas Newman, Rachel Newport, Claire Newsam, Christopher Newson, Maria Newton, Anthony YKC Ng, Ka Wing Ng, Maxine Ng, Sarah Ng, Wee Jin Ng, Thomas Ngan, Gabriel CE Ngui, Alice Ngumo, Caoimhe Nic Fhogartaigh, Nathalie Nicholas, Philip Nicholas, Rachel Nicholas, Teresa Nicholas, Donna Nicholls, Lisa Nicholls, Alice Nicholson, Anne Nicholson, Annette Nicholson, Janet Nicholson, Ian Nickson, Eileen Nicol, Elizabeth Nicol, Rebecca Nicol, Pantelis Nicola, Antony Nicoll, Pantzaris Nikolaos, Georgii Nikonovich, Annette Nilsson, Kofi Nimako, Louise Nimako, Camus Nimmo, Preethy Ninan, Mahesh Nirmalan, Muhammad Nisar, Toby Nisbett, Aksinya Nisha James, Sabaahat Nishat, Tomoko Nishiyama, Sara Nix, Jennifer Nixon, Maxine Nixon, Khwaja Nizam Ud Din, Maria Nizami, Josephine Nnadi, Lyrics Noba, Harriet Noble, Hsu Noe, Jerry Nolan, Zahid Noor, Zaid Noori, Jamie Norgrave, Louis Norman, Rachel Norman, Karen Norris, Lillian Norris, Sally Ann Nortcliffe, Fiona North, Julie North, Thomas North, Julie Northcote, John Northfield, Samantha Northover, Jurgens Nortje, Donna Norton, Rowen Norton, Holly Notman, Khalid Nourein, Timea Novak, Tony Noyce, Alan Noyon, Arlene Nubi, Mohamed Nugdallah, Anne Marie Nugent, Justine Nugent, Kribashnie Nundlall, Kieran Nunn, Michelle Nunn, Jane Nunnick, Yvonne Nupa, Zubeir Nurgat, Kelly Nwankiti, Eugenia Nweje, Godfrey Nyamugunduru, Maggie Nyirenda, Kerry Nyland, Daire O Shea, Ruth O'Donnell, Chloe O'Hara, Kevin O'Reilly, William O'Rourke, Caroline Oakley, Begho Obale, Clements Oboh, Andrew O'Brien, Clare O'Brien, Julie O'Brien, Kirsty O'Brien, Linda O'Brien, Marese O'Brien, Neale O'Brien, Rachel O'Brien, Sarah O'Brien, Tracey O'Brien, Emma O'Bryan, Ross Obukofe, Christopher O'Callaghan, Lorcan O'Connell, Maria O'Connell, Tadg OConnor, Chris O'Connor, Grainne O'Connor, Miranda Odam, Sam Oddie, Sharon Oddy, Rosie O'Dea, Yejide Odedina, Krishma Odedra, Sven W Odelberg, Natasha Odell, Omolola Oderinde, Jessica Odone, Catherine O'Donovan, Dapo Odumeru, Stephen O'Farrell, Pamela Offord, Tanwa Ogbara, Catherine Ogilvie, Ciaran O'Gorman, Oluwatomilola Ogunkeye, Udeme Ohia, Shinjali Ohja, Ohiowele Ojo, Mark O'Kane, Tolu Okeke, Eleanor OKell, Alicia Okines, Iheoma Okpala, Ernest Okpo, Maryanne Okubanjo, Ché Okyne-Turkson, Raphael Olaiya, Tim Old, Jane Oldham, Gregory Oleszkiewicz, Marta Oliveira, Annie Oliver, Catherine Oliver, Jesse Oliver, Martyn Oliver, Zoe Oliver, Nurudeen O Olokoto, Folusho Olonipile, Olumide Olufuwa, Olatomiwa Olukoya, Akinlolu Oluwole-Ojo, Laura O'Malley, Maryam Omar, Zohra Omar, Nimca Omer, Abi Omojola, Helen Omuco, Bronagh O'Neill, Connaire O'Neill, Lauran O'Neill, Chon Sum Ong, Chidera Onyeagor, Huah C Ooi, Amin Oomatia, Maria Opena, Richard Oram, Chloe Orchard, Christy Ord, Jonathan Ord, Charlotte O'Reilly, Lola Orekoya, Devaki O'Riordan, Sean O'Riordan, Amy Orme, Hannah Orme, Dave Ormrod, Charlotte Orr, Sarah Orr, Christopher Orton, Anna Osadcow, Rawlings Osagie, Rostam Osanlou, Lynn Osborne, Nigel Osborne, Rebecca Osborne, Wendy Osborne, William Osborne, Charles Osbourne, Jennifer Osei-Bobie, Mandy O'Shea, Joseph Osman, Wa'el Osman, Bashir Osman, G Osoata, Marlies Ostermann, Eoin O'Sullivan, Susan O'Sullivan, Noor Otey, Otheroro K. Otite, Marie O'Toole, Natalie Outten, Rachel Owen, Stephanie Owen, Emma Owens, Susan Owens, Yetunde Owoseni, Michael Owston, Ruth Oxlade, Feray Ozdes, Jamie Pack, Sophie Packham, Piotr Paczko, Grace Padden, Anand Padmakumar, Iain Page, Nickolas Page, Valerie Page, Jodi Paget, Katherine Pagett, Lee Paisley, Susie Pajak, Angela Pakozdi, Soubhik Pal, Sushi Pal, April Palacios, Vishnu B Palagiri Sai, Vadivu Palaniappan, Priya Palanivelu, Adrian Palfreeman, Shehan Palihavadana, Deepshikha Palit, Alistair Palmer, Lorna Palmer, Lynne Palmer, Ian Pamphlett, Anmol Pandey, Nithya Pandian, Krishnaa Pandya, Tej Pandya, Alice Panes, Yee Wei Pang, Laura Pannell, Kanwar Pannu, Sathianathan Panthakalam, Charles T Pantin, Norman Pao, Helen Papaconstantinou, Padmasayee Papineni, Marina Pappa, Kitty Paques, Kerry Paradowski, Vinay Parambil, Supathum Paranamana, Siddhant Parashar, Ian Parberry, Ana Parejasanchez, Amy Parekh, Dhruv Parekh, Louise Parfitt, Helen Parfrey, Omi Parikh, Gemma Parish, John Park, Liz Park, Angela Parker, Ben Parker, Carmel Parker, Emma Parker, Fiona Parker, Jacob Parker, Julie Parker, Laura Parker, Lucy Parker, Sara Parker, Sean Parker, Tina Parker, Kirstin Parkin, Anna Parkinson, Jill Parkinson, Lisa Parkinson, Valerie Parkinson, Chetan Parmar, Mamta Parmar, Viraj Parmar, Victoria Parris, Helen C Parry, Siobhan Parslow-Williams, Maria Parsonage, Abigail Parsons, Penny Parsons, Sarah Parsons, Richard Partridge, Kevin Parvin, Kirsten Pass, Lauren Passby, Juan Pastrana, Mital Patal, Sarah Patch, Aamie Patel, Alkesh Patel, Amisha Patel, Dakshesh Patel, Darshna Patel, Hemani Patel, Jaymik Patel, Kamal Patel, Kayur Patel, Kiran Patel, Krish Patel, Manish Patel, Martyn Patel, Mehul Patel, Naleem Patel, Nehalbhai Patel, Prital Patel, Priti Patel, Rebecca Patel, Saagar Patel, Soonie Patel, Trishna Patel, Vishal Patel, Sangeeta Pathak, Nazima Pathan, Alexandra Patience, Donna Patience, Abigail Patrick, Georgie Patrick, Jean Patrick, Simon Patten, Ben Pattenden, Ann Patterson, Charlotte Patterson, Linda Patterson, Molly Patterson, Pauline Patterson, Robert Patterson, Daniel Paul, Janice Paul, Roshni Paul, Leigh Pauls, Stephane Paulus, Amelia Pavely, Susan Pavord, Brendan Payne, David Payne, Elizabeth Payne, Ruth Payne, Tammy Payne, Abby Peacock, Linda Peacock, Louise Peacock, Sarah Peacock, Henry Peake, Rupert Pearse, Andrew Pearson, Daniel Pearson, Harriet Pearson, Karen Pearson, Kirsty Pearson, Samuel A Pearson, Sandra Pearson, Alice Peasley, Hilary Peddie, Russell Peek, Claire Pegg, Suzannah Peglar, Benjamin H Peirce, Claire Pelham, Belinda Pelle, Abigail Pemberton, Melchizedek Penacerrada, Anthony Pender, Carmel Pendlebury, Jessica Pendlebury, Sarah Pendlebury, Rachel Penfold, William Penlington, Catherine Penman, Julie Penman, Rachel Penman, Justin Penner, Kristi Penney, Alistair Penny, James Penny, Justin Pepperell, Huw Peregrine, Adriana Pereira, Ana Pereira, Rita Pereira, Carlota Pereira Dias Alves, Parmi Perera, Elena Perez, Jane Perez, Tanaraj Perinpanathan, Lakshmi Periyasamy, Francesca Perkins, Rachel Perkins, Elizabeth Perritt, Alison Perry, Emily Perry, Meghan Perry, Terrie Perry, Thomas M Perumpral, Guilherme Pessoa-Amorim, Ruth Petch, Lionel Peter, Cecilia Peters, Craig Peters, Jayne Peters, Mark Peters, Steve Peters, Tim Peters, Remy Petersen, Alexandra Peterson, Leon Peto, Iulia Petras, Ilianna Petrou, Boyanka Petrova, Mirela Petrova, Paul Pfeffer, Mysore Phanish, Paul Phelan, Christopher Philbey, Jennifer Philbin, Neil Philips, Alex Phillips, Danielle Phillips, Dylan Phillips, Karen Phillips, Pat Phillips, Rachael Phillips, Katherine Philpott, Marie Phipps, Virach Phongsathorn, Mandeep Phull, Masroor M Phulpoto, Myat TT PI, Sara Pick, James Pickard, Charlotte Pickering, Gillian Pickering, Thomas Pickett, Hayleah Pickford, Joanna Pickles, Benjamin Pickwell-Smith, Natalia Pieniazek, Charlie Piercy, Angelo Pieris, Samia Pilgrim, Paul Anthony Pillai, Zoe Pilsworth, Heather Pinches, Stacey Pinches, Julia Pinder, Kirsty Pine, Muni Tejha Pinjala, Stefania Pintus, Graeme Piper, James Piper, Richard Pipes, Tasneem Pirani, Marcus Pittman, Sally Pitts, Nicolene Plaatjies, Aiden J Plant, Naomi Platt, Robert Pleass, Rutger Ploeg, Laura Plummer, Charles Plumptre, Jonathan Pobjoy, Tatiana Pogreban, Stephen Poku, David Poles, Rachel Pollard, Louisa Pollock, Oluwamayowa Poluyi, Gary John Polwarth, Fiona Pomery, Ponmurugan Ponnusamy, Suresh Ponnusamy, Aravind Ponnuswamy, Inês Ponte Bettencourt dos Reis, Suman Pooboni, Alice Poole, Christopher Poole, Lorraine Poole, Lynda Poole, Michele Poole, Sharon Poon, Tajinder Poonian, Ella Poppitt, Christopher Porada, David Porter, Jo Porter, Linda Porter, Ross Porter, Kelly Postlethwaite, Narayana Pothina, Priyadarshan Potla, Dorota Potoczna, Jason Pott, Alison Potter, Jean Potter, Kenzi Potter, Sarah Potter, Tracey Potter, Elspeth Potton, Joanne B Potts, Julie Potts, Kathryn Potts, Una Poultney, Katherine Poulton, Vanessa Poustie, Geneen Powell, James Powell, Jordan Powell, Deborah Power, Nick Power, Gillian Powter, Joseph Poxon, Robin Poyner, Vidushi Pradhan, Helena Prady, Aalekh Prasad, Krishna Prasad, Maanvi Prasad, Fredy Prasanth Raj, Sangeetha Prasath, Sindhuja Pratheepkumar, Anezka Pratley, Steven Pratt, David Preiss, Claire Prendergast, Lynn Prentice, Peter Prentice, Verity Prescott, Laura Presland, Catharine Prest, Stephen Preston, Martha Pretorius, Natalie Prevatt, Sandra Prew, Ashley Price, Carly Price, Claire Price, David Price, Elizabeth Price, Nathan Price, Vivien Price, Nicole Priddee, Anne Priest, Kate Priestley, Jimena Prieto, Lorraine Primrose, Clare Prince, Judith Prince, Laura Prince, Janet Pring, Shirley Pringle, Veronika Pristopan, Kelly Pritchard, Lucy Pritchard, Rhys Pritchard, Simon Pritchard, Verma Priyash, Andrew Procter, Clare Proctor, Rebecca Proudfoot, Ben Prudon, David Pryor, Solomon Pudi, Joanne Pugh, Lawrence Pugh, Mark T Pugh, Nichola Pugh, Richard Pugh, Veronika Puisa, Kirandip Punia, Saleel Punnilath Abdulsamad, Laura Purandare, Claire Purcell, Corrina Purdue, Bally Purewal, Molly Pursell, Gregory Purssord, Sarah Purvis, Kathryn Puxty, Zoe Puyrigaud, Michael Pynn, Tariq Qadeer, Mohammad Qayum, Corrine Quah, Sheena Quaid, Nathaniel Quail, Charlotte Quamina, Donna Quashie, Alice Quayle, Eleanor Quek, Siobhan Quenby, Xinyi Qui, Vanessa Quick, Julie Quigley, Juan-Carlos Quijano-Campos, Andrew Quinn, Tom Quinn, Amy Quirk, Quratulain Quratulain, Danya Qureshi, Ehsaan Qureshi, Hafiz Qureshi, Hasanain Qureshi, Khadija Qureshi, Nawaz Qureshi, Qurratulain Qurratulain, Saad Qutab, Muhammad S Rabbani, Syed Rabbani, Wayne Rabin, Simon Rabinowicz, Madalina Raceala, Raissa Rachman, Laura Rad, Jane Radford, Liz Radford, Jayachandran Radhakrishnan, Cecillia Rafique, Jethin Rafique, Muhammad Rafique, Ravi Ragatha, Aiswarya Raghunathan, Abigail Raguro, Shankho D Raha, Sana Rahama, Karen Rahilly, Faisal Rahim, Abdul H Rahimi, Haseena R Rahimi, Muhammad Rahman, Salim Ur Rahman, Sharon Rainey, Lenka Raisova, Arjun Raj, Pradeep Rajagopalan, Nithy Rajaiah, Arvind Rajasekaran, Aylur Rajasri, Thurkka Rajeswaran, Jyothi Rajeswary, Jeyanthy Rajkanna, Gayathri Rajmohan, Ruth Rallan, David Ralphs, Katherine Ralston, Maximilian Ralston, Matsa Ram, Balaji Ramabhadran, Fathima Ramali, Mohamed Ramali, Athimalaipet Ramanan, Shashikira Ramanna, Maheshi Ramasamy, Dhanishta Ramdin, Jozel Ramirez, Mylah Ramirez, Geshwin Ramnarain, Lidia Ramos, Shanthi Ramraj, Alex Ramshaw, Aleem Rana, Ghulam F Rana, Rehman Rana, Abby Rand, James Rand, Helen Randall, Harpal Randheva, Poonam Ranga, Manmeet Rangar, Harini Rangarajan, Sameer Ranjan, Poormina Ranka, Rajesh Rankhelawon, Haley Ranton, Anita Rao, Sandhya Rao, Sanjay Rao, Deepak Rao, Anuja Rasarathnam, Althaf Abdul Rasheed, Alia Rashid, Khalid Rashid, Simbisai Ratcliff, Sam Ratcliffe, Sophy Ratcliffe, Sanjeev Rath, Mohmad I Rather, Selina Rathore, Aravinden Ratnakumar, Jonathan Ratoff, Deepa Rattehalli, Jason Raw, Hywel Rawlins, Gautam Ray, Michelle Ray, Adam Raymond-White, Dana Raynard, Benjamin Rayner, Nicola Rayner, Amy Raynsford, Salman Razvi, Zarine Razvi, Kerry Read, Sarah Read, Ajay Reddy, Anvesh Reddy, Harsha Reddy, Radhika Reddy, Ravi Reddy, Aine Redfern-Walsh, Alex Redome, Joan Redome, Michelle Reece, Anna Reed, John Reed, Andrew Rees, Grace Rees, Ian Rees, James Rees, Martyn Rees, Sarah Rees, Stephanie Rees, Tabitha Rees, Fiona Regan, Karen Regan, Susan Regan, Kanchan Rege, Ahmed Rehan, A Rehman, Shoib Rehman, Zainab Rehman, Ada Reid, Andrew Reid, Jennifer Reid, Jeremy Reid, Sharon Reid, Mkyla Reilly, Robert Reilly, Shonagh Reilly, Christina Reith, Alda Remegoso, Dinakaran Rengan, Stephen Renshaw, Remya Renu Vattekkat, Henrik Reschreiter, Mark Revels, Glynis Rewitzky, Charles Reynard, Dominic Reynish, Peter Reynolds, Piero Reynolds, Jonathan Rhodes, Naghma Riaz, Emily Rice, Matthew Rice, Emily Rich, Mel Rich, Alison Richards, Debbie Richards, Emma Richards, Liz Richards, Patricia Richards, Suzanne Richards, Carol Richardson, Celia Richardson, Eric Richardson, Julie Richardson, Neil Richardson, Nicky Richardson, Peter Richardson, Joanne Riches, Katie Riches, Leah Richmond, Ruth Richmond, William Ricketts, Hannah Rickman, Jonathan Ricks, Anna Riddell, Mohamed Ridha, Carrie Ridley, Paul Ridley, Gudrun Rieck, Linsey Rigby, Stephen Rigby, Hannah Riley, Matthew Riley, Phil Riley, Zwesty V P Rimba, Dominic Rimmer, Robert Rintoul, Andrew Riordan, David Ripley, Gareth Ripley, Naomi Rippon, Clive Risbridger, Chloe Rishton, Michael Riste, David Ritchie, Jane Ritchie, Andy Ritchings, Pilar Rivera Ortega, Vanessa Rivers, Batool Rizvi, Syed AS Rizvi, Syed HM Rizvi, James Robb, Matthew Robb, David Roberts, David J Roberts, Ian Roberts, Jane Roberts, Jean Roberts, Karen Roberts, Leanne Roberts, Mark Roberts, Nicky Roberts, Philip Roberts, Rebecca Roberts, Calum Robertson, Doug Robertson, James Robertson, Jamie Robertson, Nichola Robertson, Stuart Robertson, Nicole Robin, Caroline Robinson, Emma Robinson, Gisela Robinson, Hannah Robinson, Jemima Robinson, Kate Robinson, Katie Robinson, Matthew Robinson, Ryan Robinson, Sandra Robinson, Stacey Robinson, Steve Robson, Lisa Roche, Samantha Roche, Jennifer Rock, Natalie Rodden, Alistair Roddick, Jack Roddy, Marion Roderick, Alison Rodger, Faye Rodger, Megan Rodger, Alicia Rodgers, Deirdre Rodgers, Natasha Rodgers, Penny Rodgers, Vanessa Rodrigues, Rocio Rodriguez-Belmonte, Nicholas Roe, Charles Roehr, Gill Rogers, Jason Rogers, Joanne Rogers, John Rogers, Leigh Rogers, Lindsay Rogers, Louise Rogers, Michaela Rogers, Paula Rogers, Susan Rogers, Thomas Rogers, Paula Rogers, Sakib Rokadiya, Lee Rollins, Jennifer Rollo, Catherine Rolls, Atal Roman, Claire Rook, Rashmi Rook, Kevin Rooney, Lynsey Rooney, Gemma Roper, Lace P Rosaroso, Alastair Rose, Annie Rose, David Rose, Rachel Rose, Steve Rose, Vikkie Rose, Zoe Rose, Josephine Rosier, Jack Ross, Jenny Rossdale, Andrew Ross-Parker, Alex Rothman, Joanne Rothwell, Lindsay Roughley, Kathryn Rowan, Neil Rowan, Stephen Rowan, Scott Rowan-Ferry, Anna Rowe, Louise Rowe-Leete, Benjamin Rowlands, Megan Rowley, Aparajita Roy, Subarna Roy, Anna Roynon-Reed, Sam Rozewicz, Alison Rudd, Anna Rudenko, Senthan Rudrakumar, Banu Rudran, Shannon Ruff, Prita Rughani, Sharon Rundell, Jeremy Rushmer, Rosemary Rushworth, Darren Rusk, Peter Russell, Richard Russell, Cristina Russo, Marieke Rutgers, Aidan Ryan, Brendan Ryan, Lucy Ryan, Matthew Ryan, Pat Ryan, Phil Ryan, Declan Ryan-Wakeling, Nick Rylance, M Saad, Javeson Sabale, Suganya Sabaretnam, Umar Sabat, Noman Sadiq, Emma Sadler, Maria-Isabel Saez-Garcia-Holloway, Ashiq Saffy, Beth Sage, Harkiran Sagoo, Sobia Sagrir, Rajnish Saha, Sian Saha, Nikhil Sahdev, Sarvjit Sahedra, Jagdeep Sahota, Nooria Said, Sreekanth Sakthi, Hikari Sakuri, Murthy Saladi, Abdul Salam, Sofiyat Salawu, Armorel Salberg, Erika Salciute, Gina Saleeb, Mumtaz Saleh, Hizni Salih, Laylan Salih, Sarah Salisbury, SiteEneye Saliu, Rustam Salman, Angela Salmon, Jenny Salmon, Nichola Salmons, Dario Salutous, Mfon Sam, Sally Sam, Tinashe Samakomva, Renaldo Samlal, Emily Sammons, David Sammut, Mark Sammut, Zoe Sammut, Sunitha Sampath, Claire Sampson, Julia Sampson, Aashna Samson, Anda Samson, Johnson Samuel, Lorraine Samuel, Merna Samuel, Reena Samuel, Thomas DL Samuel, Younan Samuel, Elsward Samuels, Theo Samuels, Joanna Samways, Manjula Samyraju, Francisco San Diego, Ilves Sana, Veronica Sanchez, Amada Sanchez Gonzalez, Alina Sanda-Gomez, Paul Sandajam, Peter Sandercock, Amy Sanderson, Colleen Sanderson, Tom Sanderson, Kuljinder Sandhu, Loveleen Sandhu, Sam Sandow, Victoria Sandrey, Sarah Sands, Mirriam Sangombe, Mathew Sanju, Filipa Santos, Rojy Santosh, Jayanta Sanyal, Aureo F Sanz-Cepero, Dinesh Saralaya, Arun Saraswatula, Joshua Sarella, Avishay Sarfatti, Rebecca Sargent, Beatrix Sari, Khatija Sarkar, Rahuldeb Sarkar, Sruthi Sarma, Zainab Sarwar, Thea Sass, Sonia Sathe, Sobitha Sathianandan, Abilash Sathyanarayanan, Lavanya SJP Sathyanarayanan, Thozhukat Sathyapalan, Prakash Satodia, Vera Saulite, Andrew Saunders, Rachel Saunders, Samantha Saunders, Anne Saunderson, Heather Savill, Karishma Savlani, Gauri Saxena, Matthew Saxton, Amrinder Sayan, Diane Scaletta, Marta Scally, Deborah Scanlon, Jeremy Scanlon, Lyndsay Scarratt, Sean Scattergood, Alvin Schadenberg, Wendy Schneblen, Rebecca Schofield, Samuel Schofield, David Scholes, Karen Scholes, Alex Schoolmeesters, Natasha Schumacher, Nicola Schunke, Martin Schuster Bruce, Karin Schwarz, Antonia Scobie, Tim Scorrer, A. Scott, Alistair Scott, Anne Scott, Catherine Scott, Christine Scott, Emily Scott, Graham Scott, Kathyn Scott, Leanne Scott, Martha Scott, Michelle Scott, Stephen Scott, Timothy Scott, Sarah Scourfield, Wendy Scrase, Angela Scullion, Therese Scullion, Richard Seabury, Emily Seager, Cathy Seagrave, Deborah Seals, Rebecca Seaman, Eleanor Sear, Isabella Seaton, Anna Seckington, Joanna Sedano, Deborah Seddon, Gabrielle Seddon, Muhammad A Seelarbokus, Christopher Sefton, Matias Segovia, Fatima Seidu, Gillian Sekadde, Mallika Sekhar, Faye Selby, Georgina Selby, Jo Sell, Claire Sellar, Katharine Sellers, Joseph Selley, Victoria Sellick, Gobika Selvadurai, Brintha Selvarajah, Haresh Selvaskandan, Subothini S Selvendran, Gary Semple, Nandini Sen, Seema Sen, Aditya Sengupta, Niladri Sengupta, Helen Senior, Peter Senior, Susana Senra, HoJan Senya, Niranjan Setty, Abigail Seward, Teswaree Sewdin, Jack Seymour, Hussam Shabbir, Tracey Shackleton, Fiona Shackley, Tariq Shafi, Aashni Shah, Ahmar Shah, Anand Shah, Bhavni Shah, Momin Shah, Neil Shah, Pallav Shah, Priyank Shah, Qasim Shah, Sarfaraz H Shah, Snehal Shah, Suraj Shah, Syed Shah, Wajid Shah, Saarma Shahad, Sousan Shahi, Sipan Shahnazari, Muhammad Shahzeb, Aisha Shaibu, Zara Shaida, Amina Y Shaikh, Maliha Shaikh, Rajit Shail, Mariya Shaji, Muhammad Shakeel, Korah Shalan, Nadia Shamim, Kazi Shams, Alison Shanahan, Thomas Shanahan, Shaminie Shanmugaranjan, Hamed Sharaf, Muhammad Sharafat, Asir Sharif, Ajay Sharma, Akhilesh Sharma, Ash Sharma, Bhawna Sharma, Mona Sharma, Ojasvi Sharma, Poonam Sharma, Rajeev Sharma, Sanjeev Sharma, Sarkhara Sharma, Shriv Sharma, Sonal Sharma, Alexander Sharp, Charles Sharp, Gemma Sharp, Paula Sharratt, Phoebe Sharratt, Katherine Sharrocks, Emma Sharrod, Christopher Shaw, Daisy Shaw, David Shaw, Deborah Shaw, Joanne Shaw, Jonathan Shaw, Lisa Shaw, Tomos G Shaw, Anna Shawcross, Jill Shawe, Lou Shayler, Khuram Shazad, Sophy Shedwell, Jonathan Sheffield, Zak Shehata, Arshiya Sheik, Asif Sheikh, Noorann Sheikh, Laura Sheldon, Benjamin Shelley, Sarah Shelton, Anil Shenoy, Julie Shenton, Amy Shepherd, Kate Shepherd, Lorna Shepherd, Scott Shepherd, Rhian Sheppeard, Helen Sheridan, Ray Sheridan, Samuel Sherridan, Leanne Sherris, Susanna Sherwin, Shaad Shibly, Roger Shiers, Chiaki Shioi, Anand Shirgaonkar, Kim Shirley, Adebusola Shonubi, Angela Short, Richard Shortland, Rob Shortman, Rohan Shotton, Sarah Shotton, Ervin Shpuza, Nora Shrestha, Karen Shuker, Jack Shurmer, Gilbert Siame, Loria Siamia, Zanele Sibanda, Claire Sidaway, Seshnag Siddavaram, Nasir Siddique, Sohail Siddique, Nyma Sikondari, Claudia Silva Moniz, Mike Silverstone, Malcolm Sim, Theresa Simangan, Vimbai Simbi, Robert Sime, Oliver Simmons, Richard Simms, Merritt Simon, Natalie Simon, Angela Simpson, Anna Simpson, Danny Simpson, Georgina Simpson, Joanne Simpson, John Simpson, Kerry Simpson, Phillip Simpson, Thomas Simpson, Andrew Simpson, Kathryn Simpson, Cindy Sing, Ankita Singh, Claire Singh, Jayaprakash Singh, Jyoti Singh, Lokeshwar Singh, Manjeet Singh, Nadira Singh, Pankaj Singh, Prabhsimran Singh, Salil Singh, Saurabh Singh, Parag Singhal, Bryan Singizi, Manas Sinha, Utkarsh Sinha, Guy Sisson, Sarah Sithiravel, Karthikadevi Sivakumar, Shanmugasundaram Sivakumar, Darsh Sivakumran, Sivanthi Sivanadarajah, Pasupathy-Rajah Sivasothy, Rebecca Sivers, Alison Sivyer, Nicole Skehan, Robert Skelly, Orlagh Skelton, Imogen Skene, Michael J Skill, Denise Skinner, Tabitha Skinner, Victoria Skinner, Agnieszka Skorko, Iwona Skorupinska, Mariola Skorupinska, Amy Slack, Katie Slack, Wendy Slack, Heather Slade, Helen Slade, Mark Slade, Helen Slater, Lynda Slater, Nicola Slawson, Andrew Sloan, Brendan Sloan, Derek Sloan, Geraldine Sloane, Benjamin Small, Ellen Small, Emma Small, Samuel Small, Karen D Smallshaw, Andy Smallwood, Carien Smit, Aileen Smith, Alex Smith, Amanda Smith, Amy Smith, Andrew Smith, Anna Smith, Camilla Smith, Catherine Smith, Chris Smith, Christopher Smith, Dominic Smith, Eleanor Smith, Harriet Smith, Hazel Smith, Helen Smith, Jacky Smith, Janice Smith, Jessica Smith, Juliet Smith, Karen Smith, Kate Smith, Kathryn Smith, Katie Smith, Kelly Smith, Kerry Smith, Lara Smith, Linda Smith, Lisa Smith, Loren Smith, Maria Smith, Mel Smith, Oliver Smith, Rachel Smith, Rebecca Smith, Richard Smith, Ruth Smith, Sally Smith, Samantha Smith, Stacey Smith, Stephanie Smith, Susan Smith, Imogen Smith, John Smith, Gemma Smithson, Sue Smolen, Sara Smuts, Naoise Smyth, Annette Snell, David Snell, Luke Snell, Beng So, Michelle Soan, Toluleyi Sobande, Alberto Sobrino Diaz, Basit Sohail, Bina Sohail, Herminder Sohal, Roy Soiza, Mary Sokolowski, Olajumoke Solademi, Krishma Solanki, Babak Soleimani, Amanda Solesbury, Reanne Solly, Louise Solomon, Subash Somalanka, Chandrashekaraiah Somashekar, Raj Sonia, Shiu-Ching Soo, Deepti Sood, Pavandeep Soor, Germanda Soothill, Jennifer Soren, Youssef Sorour, Apina Sothinathan, Pragalathan Sothirajah, Najwa Soussi, Donna Southam, David Southern, Iain Southern, Louise Southern, Sara M Southin, Jessica Southwell, Thomas Southworth, Jason Sowter, Claudia Spalding, Enti Spata, Katie Spears, Mark Spears, John Spence, Michelle Spence, Branwell Spencer, Gisele Spencer, Sue Spencer, Tom Spencer, Helen Spicer, Rose Spicer, Helen Spickett, Jennifer Spillane, William Spiller, Kerry Spinks, Michelle Spinks, Nick Spittle, Johanna Sporrer, Karen Spreckley, Janet Spriggs, Oliver Spring, Scott Springworth, Gemma Squires, Jack Squires, Rebecca Squires, Ram Sreenivasan, K Sri Paranthamen, Ramesh Srinivasan, Asha Srirajamadhuveeti, Vino Srirathan, Chloe Stacey, Sybil Stacpoole, Louise Stadon, Tony Staincliffe, Jocasta Staines, Nikki Staines, Katie Stammers, Roxana Stanciu, Grazyna Stanczuk, Helen Stannard, Edward Stanton, Robyn Staples, Simon Stapley, Natalie Staplin, Adam Stark, Michelle Starr, Julie Staves, Rached Stead, Anthea Steel, Charlotte Steel, Conor Steele, John Steer, Vergnano Stefania, Paula Stefanowska, Katie Steinert, Caroline Stemp, Alison Stephens, David Stephensen, Elaine Stephenson, Monique Sterrenburg, Georgia Stevens, Guy Stevens, Melanie Stevens, Will Stevens, Amy Stevenson, Andrew Stevenson, Elaine Stevenson, Lesley Stevenson, Sarah Stevenson, Amanda Stewart, Claire Stewart, Colin Stewart, McKenna Stewart, Rachel Stewart, Rebecca Stewart, Richard Stewart, Jo Stickley, Gemma Stiller, Robert Stirk, Sarah Stirrup, Sarah Stock, Alexander Stockdale, Lynne Stockham, Paul Stockton, Emma Stoddard, Chris Stokes, Ben Stone, Roisin Stone, Sarah Stone, Imogen Storey, Kim Storton, Frederick Stourton, Angela Strachan, Catherine Strait, Ellen Strakosch, Emma Stratton, Jane Stratton, Sam Straw, Luke Streeter, Dieter Streit, Emma Stride, Sally Stringer, Sophia Strong-Sheldrake, Siske Struik, Carmel Stuart, Anna Stubbs, Harrison Stubbs, Ann Sturdy, Sharon Sturney, Matt Stuttard, Cristina Suarez, Karuna Subba, Christian P Subbe, Manjula Subramanian, Venkatram Subramanian, Chinari Subudhi, Rebecca Suckling, Srivatsan Sudershan, Lee Sudlow, Gayle Sugden, Peter Sugden, Rudresh Sukla, Ali Suliman, Fatimah Suliman, Ian Sullivan, Sugrah Sultan, Jennifer Summers, Mark Summerton, Samyukta Sundar, Reka Sundhar, Edmond Sung, Nadia Sunni, Jay Suntharalingam, Amitava Sur, Dharmic Suresh, Shilpa Suresh, Rachel Suri, Michael Surtees, Danielle Suter, Helen Sutherland, Rachel Sutherland, Rebecca Sutherland, Dovile Sutinyte, Deborah Sutton, John Sutton, Sam Sutton, Mihaela Sutu, Marie-Louise Svensson, Sima Svirpliene, Andrew Swain, Thomas Swaine, Christopher Swales, Lorna Swan, Nicola Swarbrick, Tirion Swart, Stephen Sweetman, Samaher Sweity, Ealish Swift, Paul Swift, Pauline Swift, Peter Swift, Rachael Swift, Rachel Swingler, Sophie Swinhoe, Katarzyna Swist-Szulik, Luke Swithenbank, Omair Syed, Catriona Sykes, Daisy Sykes, Eliot Sykes, Luke Sylvester, Dominic Symon, Andrew Syndercombe, Zoe Syrimi, Jen Syson, Gemma Szabo, Tamas Szakmany, Megan Szekely, Matthew Szeto, Maria Tadros, Amr Tageldin, Lucy Tague, Hasan Tahir, Muhammad Tahir, Silvia Taibo, Zsofia Takats, Abigail Takyi, Peter Talbot, Alison Talbot -Smith, James Talbot-Ponsonby, Richard Tallent, Bradley Tallon, Phoebe Tamblin-Hopper, Adrian Tan, Bee T Tan, Hock Tan, Huey Tan, Jade Tan, Keith Tan, WeiTeen Tan, Anand Tana, Xiaohui Tang, Christina Tanney, Tabitha Tanqueray, Emma Tanton, Ran Tao, Mark Taplin, Hayley Tarft, Priyal Taribagil, Obaid Tarin, Syed Tariq, Zeeshan Tariq, David Tarpey, Lisa Tarrant, Antonia Tasiou, Elizabeth Tatam, Margaret L Tate, Kate Tatham, Vera Tavoukjian, Alexander Taylor, Beverley Taylor, Brian Taylor, Charlie Taylor, Charlotte Taylor, David Taylor, Elisabeth Taylor, Janet Taylor, Jennifer Taylor, Joanne Taylor, Julie Taylor, Karen Taylor, Leanne Taylor, Margaret Taylor, Matthew Taylor, Melanie Taylor, Natalie Taylor, Rachael Taylor, Rachel Taylor, Samantha Taylor, Suzanne Taylor, Tina Taylor, Tracey Taylor, Vicky Taylor, Michelle Taylor-Siddons, Thomas Taynton, Amelia Te, Jessica Teasdale, Julie Tebbutt, Caroline Tee, Rajni Tejwani, Seble Tekle, Adam Telfer, Vibha Teli, Jennifer Tempany, Holly Templar, Julie Temple, Natalie Temple, Helen Tench, Yi He Teoh, Lynne Terrett, Louise Terry, Abbi Tervit, Dariusz Tetla, Kate Tettmar, Shirish Tewari, Daniel Tewkesbury, Joana Texeira, ChiaLing Tey, Clare Thakker, Manish Thakker, Amirtharajh Tharmakulasingam, Hilary Thatcher, Andrew Thayanandan, Krishna Thazhatheyil, Eaint Thein, Lambrini Theocharidou, Phyu Thet, Kapeendran Thevarajah, Mayooran Thevendra, Nang Thiri Phoo, Yvette Thirlwall, Muthu Thirumaran, Alice Thomas, Andrew Thomas, Caradog Thomas, Emma Thomas, Enson Thomas, Esther Thomas, Hannah Thomas, Helen Thomas, James Thomas, Karen Thomas, Koshy Thomas, Lucy Thomas, Rachel Thomas, Rebecca Thomas, Rhys Thomas, Ruth Thomas, Samantha Thomas, Sarah Thomas, Sherine Thomas, Tessy Thomas, Vicky Thomas, Rhian Thomas-Turner, Samantha Thomas-Wright, Catherine Thompson, Christopher Thompson, Clara Thompson, Fiona Thompson, Katharine Thompson, Laura Thompson, Liz Thompson, Luke Thompson, Michael Thompson, Orla Thompson, Rebecca Thompson, Roger Thompson, Trevor Thompson, Usilla Thompson, Nicola Thomson, Natasha Thorn, Charlotte Thorne, Nicola Thorne, Wendy Thorne, Jim Thornton, Michael Thornton, Richard Thornton, Sara Thornton, Susan Thornton, Thomas Thornton, Tracey Thornton, Allison Thorpe, Christopher Thorpe, Sarah Thorpe, Paradeep Thozthumparambil, Laura Thrasyvoulou, Hannah Thraves, Elisha Thuesday, Vicky Thwaiotes, Guy Thwaites, Simon Tiberi, Jane Tidman, Serena Tieger, Carey Tierney, Caroline Tierney, Mark Tighe, Sorrell Tilbey, Amanda Tiller, John Timerick, Elizabeth Timlick, Alison Timmis, Hayley Timms, Anne-Marie Timoroksa, Samakomva Tinashe, Heather Tinkler, Marianne Tinkler, Jacqui Tipper, Helen Tivenan, Helen T-Michael, Anne Todd, Jackie Todd, Stacy Todd, Mohamed Tohfa, Helena Tollick, Melanie Tolson, Ana Luisa Tomas, Natalia Tomasova, Sharon Tomlin, Simon Tomlins, Jo Tomlinson, James Tonkin, Ivan Tonna, Catherine Toohey, Kirsty Topham, Mathew Topping, Ruhaif Tousis, Peter Tovey, Gareth Towersey, Jason Towler, Jill Townley, Alain Townsend, Chris Townsend, Richard Tozer, Claire Tranter, Helen Tranter, Jonathan Trattles, Christopher Travill, Sarah Traynor, Karis Treuberg, Mike Trevett, Ascanio Tridente, Sanchia Triggs, Fiona Trim, Thomas Trimble, Alex Trimmings, Tom Trinick, Sven Troedson, Emily Tropman, Amy Trotter, Madeleine Trowsdale Stannard, Nigel Trudgill, Maria Truslove, Shaun Trussell, Tariq Trussell, Sara Tryon, Kyriaki Tsakiridou, Christine Tsang, Hoi Pat Tsang, Peter Tsang, Tan Tsawayo, Kyriaki Karali Tsilimpari, Georgios Tsinaslanidis, Simon Tso, Sally Tucker, Victoria Tuckley, Caroline Tuckwell, Aisha Tufail, Redmond Tully, Grace Tunesi, Saidat Turawa, Killiam Turbitt, Anna Turco, Krystyna Turek, Rezon Turel, Tolga Turgut, Claudia Turley, Alison Turnbull, Aine Turner, Ash Turner, Charlotte Turner, David Turner, Frances Turner, Gail Turner, Kate Turner, Kelly Turner, Louise Turner, Lucy Turner, Marc Turner, Mark Turner, Patricia Turner, Ruth Turner, Sally Turner, Samantha Turner, Susan Turner, Victoria Turner, Sharon Turney, Jon Turvey, Conor Tweed, David Tweed, Rebecca Twemlow, Emma Twohey, Bhavya Tyagi, Vedang Tyagi, Abigail Tyer, Jayne Tyler, Jennifer Tyler, Alison Tyzack, Petros Tzavaras, Mohammad S Uddin, Ruhama Uddin, Ruzena Uddin, Waqar Ul Hassan, Salamat Ullah, Sana Ullah, Sanda Ullah, Athavan Umaipalan, Judith Umeadi, Akudo Umeh, Wilfred Umeojiako, Ben Ummat, Charlotte Underwood, Jonathan Underwood, Laura Unitt, Adam Unsworth, Jasvinder Uppal, Veerpal S Uppal, James Uprichard, Gerry Upson, Masood Ur Rasool, Alison Uriel, Sebastian Urruela, Hiromi Uru, Miranda Usher, Rebecca Usher, Alex UsherRea, Andrew Ustianowski, Jane Uttley, Linda C Vaccari, Uddhav Vaghela, Abhay Vaidya, Bernardas Valecka, Jennifer Valentine, Balan Valeria, Pramodh Vallabhaneni, Pedro Valle Vallines, Luke Vamplew, Ekaterini Vamvakiti, Joannis Vamvakopoulos, Maud van de Venne, Alex van der Meer, Nora van der Stelt, Joseph Vance-Daniel, Rama Vancheeswaran, Caryn Vander Riet, Samuel I Vandeyoon, Padma Vankayalapati, Piyush Vanmali, Chloe Vansomeren, William Van't Hoff, Sejal Vara, Kate Vardigans, Stehen J Vardy, Anu Varghese, Maria Varghese, William Varney, Giulia Varnier, Valeria Vasadi, Olivia Vass, Vimal Vasu, Vasanthi Vasudevan, Manu Vatish, Heloyes Vayalaman, Christopher Vaz, Niki Veale, Sachuda Veerasamy, Bar Velan, Swati Velankar, Luxmi Velauthar, Neyme Veli, Nicola Vella, Anitha Velusamy, Ian Venables, Mavi Venditti, David Veniard, Ramya Venkataramakrishnan, Richard Venn, Robert Venn, Lyn Ventilacion, Joanne Vere, Mark Veres, Stefania Vergnano, Will Verling, Amit Verma, Rachel Vernall, Britney Vernon, Mark Vertue, Jerik Verula, Natalie Vethanayagam, Lucy Veys, Carinna Vickers, Saji Victor, Jennifer Vidler, Wayne Vietri, Bavithra Vijayakumar, Vinod Warrier Vijayaraghavan Nalini, Brigita Vilcinskaite, Neringa Vilimiene, Sudharkar Vimalanathan, Lynn Vinall, Sylvia Vinay, Latha Vinayakarao, Rachel Vincent, Rosie Vincent, Pritpal Virdee, Emma Virgilio, Abdullah M Virk, Elisa Visentin, Jeyakumar Visuvanathan, Karunakaran Vithian, Sorice Vittoria, Elena Vlad, Ben Vlies, Alain Vuylsteke, Eleftheria Vyras, Richard Wach, Beverley Wadams, Susan Wadd, Natalia Waddington, Kirsten Wadsworth, Syed EI Wafa, Daniel Wagstaff, Lynda Wagstaff, Dalia Wahab, Zaroug Wahbi, Abiodun Waheed Adigun, Sawan Waidyanatha, Rachel Wake, Alice Wakefield, William Wakeford, Michelle Wakelin, Fiona Wakinshaw, Andrew Walden, Jane Walden, Lorna Walding, Alexandria Waldron, Gemma Walker, Harriet Walker, Ian Walker, Jasmine Walker, Kevin Walker, Kim Walker, Linda Walker, Marie T Walker, Olivia Walker, Rachel Walker, Rebecca Walker, Susan Walker, Derek Wallbank, Rebecca Wallbutton, Jessica Wallen, Karl Wallendszus, Arabella Waller, Fiona Waller, Rosemary Waller, Gabiel Wallis, Gabriel Wallis, Louise Wallis, Donna Walsh, Elizabeth Walsh, Livia Walsh, Deborah Walstow, Daniel Walter, Alex Walters, Holt Walters, James Walters, Jocelyn Walters, Eileen Walton, Lucy Walton, Olivia Walton, Sharon Walton, Susan Walton, Mandy Wan, Thin Wan, Mary Wands, Rachel Wane, Frank Wang, Nick Wang, Ran Wang, Deborah Warbrick, Samantha Warburton, Deborah Ward, Emma Ward, Joanna Ward, Karen Ward, Luke Ward, Nicola Ward, Rachael Ward, Rebecca Ward, Thomas Ward, Tom Ward, Scott A Warden, Adele Wardle, Karen Wardle, Steve Wardle, Hassan Wardy, Scott Waring, Jenny Warmington, Ben Warner, Christian Warner, Lewis Warnock, Sarah Warran, Jade Warren, Lisa Warren, Yolanda Warren, Hannah Warren-Miell, Gill Warwick, Charlotte Washington, Helen Wassall, Hazel J Watchorn, Holly Waterfall, Abby Waters, Donald Waters, Mark Waterstone, Catherine Watkins, Catrin Watkins, Eleanor Watkins, Karen Watkins, Lynn Watkins, Nick Watkins, Abigail Watson, Adam JR Watson, Ekaterina Watson, Eleanor Watson, Paul Watson, Rebecca Watson, Robert Watson, Sandra Watson, Malcolm Watters, Donna Watterson, Daniel Watts, John Watts, Merlin Watts, Victoria Waugh, Emma Wayman, Akhlaq Wazir, Mark Weatherhead, Nick Weatherly, Paul Weaver, Hayley Webb, Kathryn Webb, Kylie Webb, Stephen Webb, Cheryl Websdale, Deborah Webster, Ian Webster, Tim Webster, Kathleen Wedgeworth, Ling Wee, Rebecca Weerakoon, Thanuja Weerasinghe, Janaka Weeratunga, Maria Weetman, Shuying Wei, Immo Weichert, Hugh Welch, James Welch, Leanne Welch, Steven Welch, Samantha Weller, Lucy Wellings, Brian Wells, Susan Wellstead, Berni Welsh, Richard Welsh, Ingeborg Welters, Rachael Welton, Lauren Wentworth, Kate Wesseldine, James Wesson, Jim Wesson, Adam West, Magdelena West, Raha West, Ruth West, Sophie West, Luke Western, Ruth Westhead, Heather Weston, Alice Westwood, Bill Wetherill, Sharon Wheaver, Helen Wheeler, Ben Whelan, Matthew Whelband, Amanda Whileman, Alison Whitcher, Abbie White, Andrew White, Benjamin White, Christopher White, Duncan White, Emily White, James White, Jonathan White, Katie White, Marie White, Nick White, Sarah White, Sonia White, Stephen White, Tracey White, Catherine Whitehead, Anne Whitehouse, Claire Whitehouse, Tony Whitehouse, Julia Whiteley, Sophie Whiteley, Victoria Whiteside, Drew Whitley, Kaitlyn Whitley, Gabriel Whitlingum, David Whitmore, Elizabeth Whittaker, Lindsay Whittam, Andrew Whittingham Hirst, Ashley Whittington, Helen Whittle, Robert Whittle, Suzanne Whyte, Eunice Wiafe, Lou Wiblin, John Widdrington, Jason Wieboldt, Hannah Wieringa, Cornelia Wiesender, Laura Wiffen, Andrew Wight, Christopher Wignall, Danielle Wilcock, Emma Wilcock, Louise Wilcox, Laura Wild, Stephen Wild, Michael Wilde, Peter Wilding, Ritchie Wildman, Tracey Wildsmith, Joe Wileman, Donna Wiles, Joy Wiles, Kate Wiles, Elva Wilhelmsen, Thomas Wiliams, Chloe Wilkes, Janet Wilkie, David Wilkin, Hannah Wilkins, Joy Wilkins, Suzanne Wilkins, Helen Wilkinson, Holly Wilkinson, Iain Wilkinson, Lesley Wilkinson, Martin Wilkinson, Nicola Wilkinson, Sophia Wilkinson, Susan Wilkinson, Tim Wilkinson, Sylvia Willetts, Aimee Williams, Alexandra Williams, Alison Williams, Angharad Williams, Ava Williams, Carl Williams, Caroline V Williams, Claire Williams, Dewi Williams, Gail Williams, Gemma Williams, Gina Williams, Hannah Williams, James Williams, Jayne Williams, Jennie Williams, John Williams, Joseph Williams, Karen Williams, Kathryn Williams, Marie Williams, Matthew Williams, Patricia Williams, Penny Williams, Rachael Williams, Rupert Williams, Samson Williams, Sarah Williams, Sophie Williams, Tamanna Williams, Annie Williamson, Cath Williamson, Catherine Williamson, Dawn Williamson, James D Williamson, Rachel Williamson, Helen Williamson, Bruce Willian, Elizabeth Willis, Emily Willis, Heather Willis, Herika Willis, Joanna Willis, Laura Willmott, Louise Wills, Lucy Willsher, Catherine Willshire, Francesca Willson, Alison Wilson, Andrea Wilson, Antoinette Wilson, Billy Wilson, Catherine Wilson, Eve Wilson, James Wilson, Karen Wilson, Kate Wilson, Lucinda Wilson, Mark Wilson, Matthew Wilson, Toni Wilson, Evie Wiltsher, Marlar Win, Tin Win, Wut Yee Win Win, Lucinda Winckworth, Laura Winder, Piers Winder, Phillip Windrum, Kerry Winham-Whyte, Helen Winmill, Simon Winn, Carmen Winpenny, Helen Winslow, Helen Winter, Jonathan Winter, Pascal Winter, Barbara Winter-Goodwin, Stephen Wisdom, Matthew Wise, Martin Wiselka, Rebecca Wiseman, Sophie Wiseman, Steven Wishart, Holly Wissett, Eric Witele, Nicholas Withers, Janet Wittes, Donna Wixted, Therese Wodehouse, Will Wolf, Nicola Wolff, Kirsten Wolffsohn, Rebecca Wolf-Roberts, Magda Wolna, Elena Wolodimeroff, Adam Wolstencroft, Alan Wong, Charlotte Wong, Chi-Hung Wong, Edwin Wong, Jessica Sue Yi Wong, Kit Y Wong, Lee Wong, Mei Yin Wong, Nick Wong, Sam Wong, Yun Man Wong, Amanda Wood, Caroline Wood, Carrie Anne Wood, Dianne Wood, Fiona Wood, Hannah Wood, Jennifer Wood, Joe Wood, Julia Wood, Kathryn Wood, Lisa Wood, Louise Wood, Michelle Wood, Stephen Wood, Tracy Wood, Ursula Wood, Katharine Woodall, Rebecca Woodfield, Christopher Woodford, Elizabeth Woodford, Jill Woodford, Luke Woodford, Louise Woodhead, Timothy Woodhead, Philip Woodland, Marc Woodman, Debra Woods, Jane Woods, Katherine Woods, Sarah Woods, Zoe Woodward, Rachel Wookey, Megan Woolcock, Gemma Wooldridge, Rebecca Woolf, Chris Woollard, Christopher Woollard, Louisa Woollen, Emma Woolley, Jade Woolley, Daniel Woosey, Dan Wootton, Joanne Wootton, Daniel Worley, Stephy Worton, Jonathan Wraight, Maria Wray, Tim Wreford-Bush, Joanne Wren, Kim Wren, Lynn Wren, Caroline Wrey Brown, Catherine Wright, Demi Wright, Francesca Wright, Imogen Wright, Lee Wright, Lianne Wright, Pete Wright, Rachel Wright, Rebecca Wright, Stephanie Wright, Tim Wright, Caroline Wroe, Hannah Wroe, Henry Wu, Peishan Wu, Pensee Wu, Jonathan Wubetu, Retno Wulandari, Craig Wyatt, Frederick Wyn-Griffiths, Inez Wynter, Bindhu Xavier, Arnold Xhikola, Zhongyang Xia, Huiyuan Xiao, Masseh Yakubi, May Yan, Freda Yang, Yingjia Yang, Michael Yanney, Woei Lin Yap, Nabil Yaqoob, Naairah Yaqub, Salima Yasmin, Bryan Yates, David Yates, Edward Yates, Helen Yates, Julie Yates, Mark Yates, Charlotte Yearwood Martin, Andrew Yeatman, Khin Yein, Fiona Yelnoorkar, Peter Yew, Kawai Yip, Laura Ylquimiche, Laura Ylquimiche Melly, Inez Ynter, H Yong, Jemma Yorke, Jasmine Youens, Abdel Younes Ibrahim, Eoin Young, Gail Young, Louise Young, Richard Young, Asfand Yousafzar, Sajeda Youssouf, Ahmed Yousuf, Chrissie Yu, Bernard Yung, Daniel Yusef, Said Yusef, Intekhab Yusuf, Anna-Sophia Zafar, Silvia Zagalo, Su Zaher, Aqsa Zahoor, Kareem Zaki, Nabhan Zakir, Kasia Zalewska, Ane Zamalloa, Mohsin Zaman, Raisa Zaman, Shakir Zaman, Julie Zamikula, Louise Zammit, Marie Zammit-Mangion, Lynn Zarb, Esther Zebracki, Daniel Zehnder, Lisa Zeidan, Marian Zelman, Xiaobei Zhao, Dongling Zheng, Doreen Zhu, Madiha Zia, Omar Zibdeh, Rabia Zill-E-Huma, Ei Thankt Zin, Veronica Zindonda, Eleanor Zinkin, Vivian Zinyemba, Christos Zipitis, Arkadiusz Zmierczak, Azam Zubir, Roslin Zuha, Naz Zuhra, Rasha Zulaikha, Sabrina Zulfikar, Carol Zullo, Ana Zuriaga-Alvaro, Will Zuurbier, Sheba Zyengi

## Abstract

**Background:**

Many patients with COVID-19 have been treated with plasma containing anti-SARS-CoV-2 antibodies. We aimed to evaluate the safety and efficacy of convalescent plasma therapy in patients admitted to hospital with COVID-19.

**Methods:**

This randomised, controlled, open-label, platform trial (Randomised Evaluation of COVID-19 Therapy [RECOVERY]) is assessing several possible treatments in patients hospitalised with COVID-19 in the UK. The trial is underway at 177 NHS hospitals from across the UK. Eligible and consenting patients were randomly assigned (1:1) to receive either usual care alone (usual care group) or usual care plus high-titre convalescent plasma (convalescent plasma group). The primary outcome was 28-day mortality, analysed on an intention-to-treat basis. The trial is registered with ISRCTN, 50189673, and ClinicalTrials.gov, NCT04381936.

**Findings:**

Between May 28, 2020, and Jan 15, 2021, 11558 (71%) of 16287 patients enrolled in RECOVERY were eligible to receive convalescent plasma and were assigned to either the convalescent plasma group or the usual care group. There was no significant difference in 28-day mortality between the two groups: 1399 (24%) of 5795 patients in the convalescent plasma group and 1408 (24%) of 5763 patients in the usual care group died within 28 days (rate ratio 1·00, 95% CI 0·93–1·07; p=0·95). The 28-day mortality rate ratio was similar in all prespecified subgroups of patients, including in those patients without detectable SARS-CoV-2 antibodies at randomisation. Allocation to convalescent plasma had no significant effect on the proportion of patients discharged from hospital within 28 days (3832 [66%] patients in the convalescent plasma group *vs* 3822 [66%] patients in the usual care group; rate ratio 0·99, 95% CI 0·94–1·03; p=0·57). Among those not on invasive mechanical ventilation at randomisation, there was no significant difference in the proportion of patients meeting the composite endpoint of progression to invasive mechanical ventilation or death (1568 [29%] of 5493 patients in the convalescent plasma group *vs* 1568 [29%] of 5448 patients in the usual care group; rate ratio 0·99, 95% CI 0·93–1·05; p=0·79).

**Interpretation:**

In patients hospitalised with COVID-19, high-titre convalescent plasma did not improve survival or other prespecified clinical outcomes.

**Funding:**

UK Research and Innovation (Medical Research Council) and National Institute of Health Research.

## Introduction

A substantial proportion of individuals with SARS-CoV-2 require hospital care, which can progress to critical illness with hypoxic respiratory failure. In patients with severe COVID-19, immunomodulation with corticosteroids and IL-6 receptor antagonists has been shown to improve survival.^1^, ^2^ Treatments that effectively inhibit viral replication might reduce tissue damage and allow time for the host to develop an adaptive immune response that can clear the infection. However, no treatment directed against the virus has been shown to reduce mortality (although remdesivir might shorten the duration of hospital stay).^3^

Humoral immunity is a key component of the immune response to SARS-CoV-2, and it matures over several weeks following infection. Anti-SARS-CoV-2 antibodies are detectable at a mean of 13 days after symptom onset, but neutralising titres do not peak until day 23, and there is wide variation in both the timing of seroconversion and peak antibody concentrations between infected individuals.^4^ Although patients with severe COVID-19 generally have higher final antibody concentrations than those with mild disease, their antibody responses are delayed.^5^ Antibodies might modulate acute viral disease either through a direct antiviral effect—by binding and neutralising free virus—or indirectly by activating antiviral pathways—such as the complement cascade, phagocytosis, and cellular cytotoxicity. Conversely, there is also a possibility that antibodies might enhance disease, either by promoting viral entry or by proinflammatory mechanisms, such as Fcγ receptor stimulation.^6^

Convalescent plasma has been used for more than 100 years as passive immunotherapy for influenza pneumonia, and more recently for SARS-CoV.^7^ Although observational studies have suggested that convalescent plasma might reduce mortality in severe viral respiratory infections evidence from randomised trials remains scarce and inconclusive.^8^ Convalescent plasma has been used widely outside of clinical trials, including by more than 100 000 patients in the US Food and Drugs Administration (FDA) Expanded Access Program.^9^ An observational analysis of 3082 patients in this programme reported that in patients who had not received mechanical ventilation, 30-day mortality was lower in those transfused with higher-titre plasma (containing higher concentrations of anti-SARS-CoV-2 spike IgG) compared with those who received lower-titre plasma.^10^ A number of randomised trials of convalescent plasma in patients hospitalised with COVID-19 have been reported, but these trials have all been small and inconclusive.^11^, ^12^, ^13^, ^14^, ^15^, ^16^, ^17^, ^18^, ^19^, ^20^ Moreover, patients who are hospitalised with COVID-19 are heterogeneous and any benefit of convalescent plasma could depend on the stage of disease, perhaps being restricted to those with milder disease early in the course of their illness or those who have not mounted an effective antibody response.^14^ Therefore, the efficacy of convalescent plasma as a treatment for patients hospitalised with COVID-19 is uncertain. We aimed to evaluate the efficacy and safety of convalescent plasma in patients hospitalised with COVID-19.

**Research in context**

**Evidence before this study**

We searched the MEDLINE, Embase, MedRxiv, and bioRxiv databases from Sept 1, 2019, to March 23, 2021, for randomised trials or meta-analyses of trials evaluating the effect of convalescent plasma in patients hospitalised with COVID-19 using the search terms (“COVID-19”, “COVID”, “SARS-CoV-2”, “2019-nCoV”, or “Coronavirus”) and (“convalescent plasma”, “hyperimmune plasma”, “immune plasma”, “passive immunization”, or “plasma therapy”). 12 trials were identified. Two trials were excluded from the meta-analysis: one trial of 49 patients that did not have robust allocation concealment and one trial of 30 patients that did not report mortality. In two trials participants and clinicians were masked to treatment allocation and the remaining eight trials were open-label. There was some concern about missing outcome data in one trial, but the remaining nine studies were assessed as having a low risk of bias when using an outcome of mortality. These trials included 1495 randomly assigned patients, of whom 218 died. Most of these studies recruited patients shortly after admission to hospital, as was the case in RECOVERY.

**Added value of this study**

RECOVERY is the largest randomised trial to report results of the effect of convalescent plasma in patients hospitalised with COVID-19. We found that compared with usual care alone, high-titre convalescent plasma did not reduce 28-day mortality, the probability of discharge within 28 days, or the probability of progressing to the composite outcome of invasive mechanical ventilation or death in patients who were not receiving invasive mechanical ventilation at randomisation. We saw no evidence of any material benefit or hazard of convalescent plasma in any patient subgroup. Taking the results of all trials together, including RECOVERY which includes about eight-times as much information as all other trials combined, allocation to convalescent plasma was associated with a mortality rate ratio 0·98 (95% CI 0·91–1·06; p=0·63).

**Implications of all the available evidence**

For patients admitted to hospital with COVID-19, convalescent plasma offers no material therapeutic benefits.

## Methods

### Study design and participants

The RECOVERY trial is an investigator-initiated, individually randomised, controlled, open-label, adaptive platform trial to evaluate the effects of potential treatments in patients hospitalised with COVID-19. Details of the trial design and results for other evaluated treatments (dexamethasone, hydroxychloroquine, lopinavir–ritonavir, azithromycin, and tocilizumab) have been published previously.^2^ The trial is underway at 177 NHS hospital organisations in the UK (appendix pp 5–28), supported by the National Institute for Health Research Clinical Research Network. The trial was coordinated by the trial sponsor, the Nuffield Department of Population Health, University of Oxford (Oxford, UK). The trial is being done in accordance with the principles of the International Conference on Harmonisation–Good Clinical Practice guidelines and approved by the UK Medicines and Healthcare products Regulatory Agency and the Cambridge East Research Ethics Committee (20/EE/0101). The protocol, statistical analysis plan, and additional information are available online and in the appendix (pp 66–151).

Hospitalised patients of any age were eligible for the trial if they had clinically suspected or laboratory-confirmed SARS-CoV-2 infection and no medical history that might, in the opinion of the attending clinician, put them at significant risk if they were to participate in the trial. Written informed consent was obtained from all patients or from their legal representative if they were too unwell or unable to provide consent.

### Randomisation and masking

Baseline data were collected using a web-based case report form that included demographics, level of respiratory support, major comorbidities, suitability of the trial treatment for a particular patient and treatment availability at the trial site (appendix pp 35–37). Patients had a serum sample taken before random assignment for the purpose of assessing the presence of antibodies against SARS-CoV-2.

Until Sept 18, 2020, eligible and consenting patients were randomly assigned (1:1) to receive either usual care (usual care group) or usual care plus convalescent plasma (convalescent plasma group). From Sept 18, 2020, patients were randomly assigned (1:1:1) to the usual care group, convalescent plasma group, or to receive usual care plus REGN-COV2 (a combination of two monoclonal antibodies directed against SARS-CoV-2 spike protein; appendix pp 35–37). The REGN-COV2 evaluation is ongoing and not reported here. Random assignment was unstratified and done by local clinical or research staff using a web-based interface with allocation concealment (appendix pp 33–34). For some patients, convalescent plasma was either declined, unavailable at the trial site at the time of enrolment, or considered in the opinion of the attending doctor to be definitely contraindicated (eg, known moderate or severe allergy to blood components). These patients were not included in the comparison of convalescent plasma versus usual care.

In a factorial design, patients could be simultaneously randomly assigned to other treatment groups: (1) hydroxychloroquine or dexamethasone or azithromycin or lopinavir–ritonavir or colchicine versus usual care, and (2) aspirin versus usual care (appendix pp 33–34). The trial also allowed a subsequent randomisation for patients with progressive COVID-19 (evidence of hypoxia and a hyperinflammatory state) to tocilizumab versus usual care. Participants and local study staff were not masked to the allocated treatment. Several of these treatment groups were added to or removed from the protocol over the period that convalescent plasma was evaluated (appendix pp 29–34). The trial steering committee, investigators, and all other individuals involved in the trial were masked to outcome data during the trial.

### Procedures

Convalescent plasma donors were recruited and screened by the four UK blood services: NHS Blood and Transplant, the Northern Ireland Blood Transfusion Service, the Scottish National Blood Transfusion Service, and the Welsh Blood Service (appendix pp 2–4, 29). Only plasma donations with a sample to cutoff ratio of 6·0 or more on the EUROIMMUN IgG enzyme-linked immunosorbent assay (ELISA) targeting the spike glycoprotein (PerkinElmer, London, UK) were supplied for the RECOVERY trial (appendix p 29). EUROIMMUN IgG has been shown to correlate well with neutralisation assays, and a sample to cutoff ratio of 6·0 or more was previously shown to be associated with neutralising titres of 1:100 or more in convalescent plasma.^21^, ^22^, ^23^, ^24^ The US FDA has determined that convalescent plasma with a EUROIMMUN sample to cutoff of 3·5 or more qualifies as high titre and can be used for the treatment of hospitalised patients under an Emergency Use Authorisation.^9^ Patients in the convalescent plasma group received two units (275 ml [200–350]) intravenously, the first as soon as possible after randomisation and the second (from a different donor) the following day and at least 12 h after the first.

Early safety outcomes were recorded by site staff using an online form 72 h after randomisation (appendix pp 38–42). An online follow-up form was completed by site staff when patients were discharged, had died, or at 28 days after randomisation, whichever occurred first (appendix pp 43–49). Information was recorded on adherence to allocated trial treatment, receipt of other COVID-19 treatments, duration of admission, receipt of respiratory or renal support, and vital status (including cause of death). In addition, routine health-care and registry data were obtained, including information on vital status at day 28 (with date and cause of death); discharge from hospital; and receipt of respiratory support or renal replacement therapy.

Baseline SARS-CoV-2 serostatus for each participant was determined using serum samples taken at the time of randomisation. Analysis was done at a central laboratory with a validated 384 well plate indirect ELISA (appendix p 29).^25^ Participants were categorised as seropositive or seronegative using a predefined assay threshold with a 99% or higher sensitivity and specificity in detecting individuals with SARS-CoV-2 infection at least 20 days previously.^25^

### Outcomes

Outcomes were assessed at 28 days after randomisation, with additional analyses specified at 6 months. The primary outcome was all-cause mortality. Secondary outcomes were time to discharge from hospital and, in patients not receiving mechanical ventilation at randomisation, subsequent receipt of invasive mechanical ventilation (including extra-corporeal membrane oxygenation) or death. Prespecified, subsidiary clinical outcomes included receipt of ventilation, time to successful cessation of invasive mechanical ventilation (defined as removal of invasive mechanical ventilation within, and survival to, 28 days), and use of renal dialysis or haemofiltration.

Prespecified safety outcomes were transfusion related adverse events at 72 h following randomisation (worsening respiratory status, suspected transfusion reaction, fever, hypotension, haemolysis, and thrombotic events), cause-specific mortality, and major cardiac arrhythmia. Information on serious adverse reactions to convalescent plasma was collected in an expedited fashion via the existing NHS Serious Hazards of Tranfusion haemovigilence scheme.

### Statistical analysis

In accordance with the statistical analysis plan, an intention-to-treat comparison was done between patients in the convalescent plasma group and those in the usual care group for whom convalescent plasma was both available and suitable as a treatment. For the primary outcome of 28-day mortality, the log-rank observed minus expected statistic and its variance were used both to test the null hypothesis of equal survival curves (ie, the log-rank test) and to calculate the one-step estimate of the average mortality rate ratio. We used Kaplan-Meier survival curves to display cumulative mortality over the 28-day period. We used similar methods to analyse time to hospital discharge and successful cessation of invasive mechanical ventilation, with patients who died in hospital right-censored on day 29. Median time to discharge was derived from Kaplan-Meier estimates. For the prespecified, composite, secondary outcome of progression to invasive mechanical ventilation or death within 28 days (in those not receiving invasive mechanical ventilation at randomisation) and the subsidiary clinical outcomes of receipt of ventilation and use of haemodialysis or haemofiltration, the precise dates were not available so the risk ratio was estimated instead.

Prespecified analyses of the primary outcome were done in seven subgroups defined by characteristics at randomisation: age, sex, ethnicity, respiratory support received, days since symptom onset, use of systemic corticosteroids, and presence of anti-SARS-CoV-2 antibody. Observed effects within these subgroup categories were compared using a χ^2^ test. Subgroup analyses according to these baseline characteristics were also done for the secondary outcomes. Post-hoc exploratory analyses of the primary outcome included examination by days since symptom onset, using four subcategories rather than the two that were prespecified, and level of respiratory support by subdividing the oxygen only group into three subcategories. In late 2020, a new SARS-CoV-2 variant, named B.1.1.7, with multiple substitutions in the receptor binding domain of the spike glycoprotein emerged in southeast England and rapidly grew to become the dominant virus variant throughout the UK.^26^ Convalescent plasma from individuals infected before the emergence of B.1.1.7 show a modest reduction in ability to neutralise B.1.1.7 compared with earlier SARS-CoV-2 virus variants.^27^ The clinical significance of this reduced in-vitro neutralisation is not known. To assess if there was evidence of a difference in the effectiveness of convalescent plasma before and after the emergence of B.1.1.7, an additional post-hoc exploratory analysis was done of the primary outcome comparing effects in patients randomly assigned before Dec 1, 2020, with those randomly assigned from Dec 1, 2020, onwards.^26^

Additional sensitivity analyses included restricting analysis of the primary outcome to patients with a positive PCR test for SARS-COV-2 and repeating subgroup analyses of the primary and secondary outcomes by presence of anti-SARS-CoV-2 antibody after adjustment for age. Age adjustment was done because in seronegative patients those assigned to the convalescent plasma group were slightly younger than those assigned to the usual care group, whereas in seropositive patients those assigned to the convalescent plasma group were slightly older than those assigned to the usual care group. A final prespecified exploratory analysis estimated whether the effect of allocation to convalescent plasma varied depending on whether the patient was simultaneously allocated azithromycin (the only other treatment that has both already reported its results and to which substantial numbers of patients could have been assigned at the same time as they were randomly assigned to receive convalescent plasma or usual care).

Estimates of rate and risk ratios are shown with 95% CIs. All p values are two-sided and are shown without adjustment for multiple testing. The full database is held by the trial team who pooled the data from trial sites and did the analyses at the Nuffield Department of Population Health, University of Oxford.

For the primary outcome of 28-day mortality, the results from RECOVERY were subsequently included in a meta-analysis of results from all previous randomised trials of convalescent plasma versus usual care in patients with COVID-19. For each trial, we compared the observed number of deaths among patients allocated convalescent plasma with the expected number if all patients were at equal risk (ie, we calculated the observed minus expected statistic [o–e], and its variance [v]). For RECOVERY, these were taken as the log-rank observed minus expected statistic and its variance but for other trials, where the exact timing of each death was not available, these were calculated from standard formulae for 2 × 2 contingency tables. We then combined trial results using the log of the mortality rate ratio calculated as the inverse-variance weighted average S/V with variance 1/V (and hence with 95% CI S/V ±1·96/√V), where S is the sum over all trials of (O–E) and V is the sum over all trials of v. Analyses were done with SAS (version 9.4) and R (version 3.4).

As stated in the protocol, appropriate sample sizes could not be estimated when the trial was being planned at the start of the COVID-19 pandemic. During the trial, external data suggested that any benefits of antibody-based therapies might be higher in patients who had not raised an adequate antibody response of their own.^14^ Consequently, while still masked to the results of the trial, the RECOVERY steering committee determined that the trial should enrol sufficient patients to provide at least 90% power at a two-sided p value of 0·01 to detect a proportional reduction in 28-day mortality of a fifth in patients with and, separately, without detectable SARS-CoV-2 antibodies at randomisation (appendix p 34).

On Jan 7, 2021, the independent data monitoring committee did a routine review of the data and recommended that the chief investigators pause recruitment to the convalescent plasma comparison in those patients receiving invasive mechanical ventilation (including extracorporeal membrane oxygenation) at the time of randomisation. At the same time, the committee recommended that recruitment to the convalescent plasma comparison continue for all other eligible patients.

On Jan 14, 2021, the data monitoring committee did another routine review of the data and notified the chief investigators that there was no convincing evidence that continued recruitment would provide conclusive proof of worthwhile mortality benefit, either overall or in any prespecified subgroup. The committee recommended that recruitment to the convalescent plasma portion of the study should cease and follow-up be completed. Enrolment of patients to the convalescent plasma comparison was closed on Jan 15, 2021, and the preliminary result for the primary outcome was made public. The trial is registered with ISRCTN, 50189673, and ClinicalTrials.gov, NCT04381936.

### Role of the funding source

The funders of the trial had no role in trial design, data collection, data analysis, data interpretation, or writing of the report.

## Results

**Figure 1:**
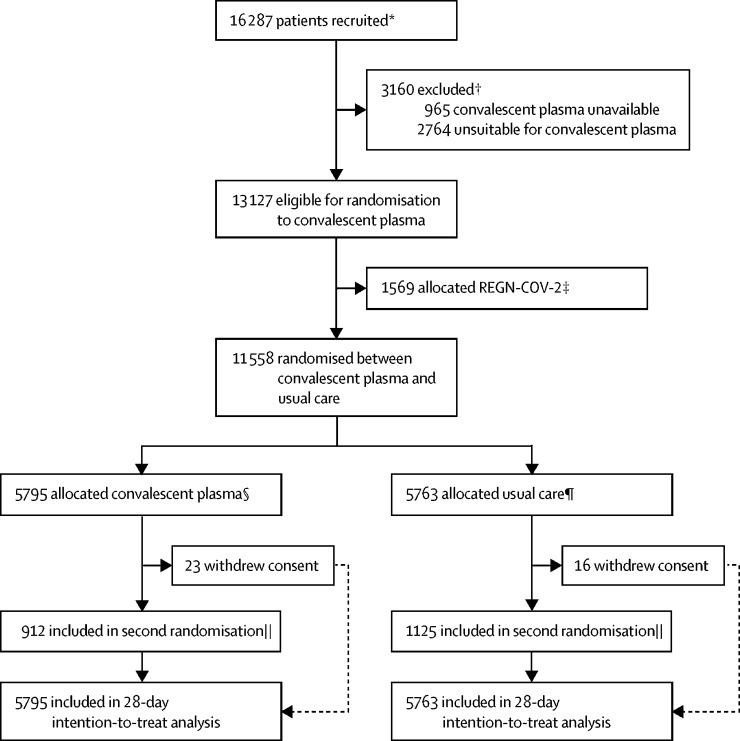
Trial profile *Number recruited overall during period that patients could be recruited into convalescent plasma comparison. †Reasons for exclusion are not mutually exclusive. ‡Patients in the group are not included in the analyses of this study. §5301 of 5795 patients with completed follow-up at time of analysis received convalescent plasma. ¶17 of 5763 patients with completed follow-up at time of analysis received convalescent plasma. ||A second randomisation to tocilizumab versus usual care in patients with hypoxia and C-reactive protein ≥75 mg/L was introduced in protocol version 4.0; 426 patients in the convalescent plasma group were randomly assigned to receive tocilizumab with 486 randomly assigned to receive usual care alone; 573 patients in the usual care group were randomly assigned to receive tocilizumab with 552 randomly assigned to receive usual care alone.

Between May 28, 2020, and Jan 15, 2021, 13127 (81%) of 16 287 patients enrolled into the RECOVERY trial were eligible to receive convalescent plasma (figure 1). 1569 (12%) were randomly assigned to the REGN-COV-2 group and are not included in the analyses reported here. Of the remaining 11558 patients, 5795 (50%) were randomly assigned to the convalescent plasma group and 5763 (50%) to the usual care group. The mean age of the patients was 63·5 (SD 14·7) years, and the median time from symptom onset to randomisation was 9 days (IQR 6–12; table 1; appendix p 51). At randomisation, 617 (5%) of 11 558 patients were receiving invasive mechanical ventilation, 10 044 (87%) were receiving oxygen only (with or without non-invasive respiratory support), and 897 (8%) were receiving no oxygen therapy (appendix p 51). 10 681 (92%) of 11 558 patients were receiving corticosteroids at time of randomisation. By chance, a slightly lower proportion of men were randomly assigned to the convalescent plasma group than the usual care group, so Cox regression analyses adjusted for sex are provided (appendix p 57), which are almost identical to the main results shown.

**Table 1:** Baseline characteristics

		**Convalescent plasma group (n=5795)**	**Usual care group (n=5763)**
Mean age, years		63·5 (14·7)	63·4 (14·6)
Age groups			
	<70*	3705 (64%)	3748 (65%)
	70–79	1310 (23%)	1281 (22%)
	≥80	780 (13%)	734 (13%)
Sex			
	Men	3643 (63%)	3787 (66%)
	Women†	2152 (37%)	1976 (34%)
Ethnicity			
	White	4493 (78%)	4421 (77%)
	Black, Asian, and minority ethnic	833 (14%)	887 (15%)
	Unknown	469 (8%)	455 (8%)
Median number of days since symptom onset		9 (6–12)	9 (6–12)
Median number of days since admission to hospital		2 (1–3)	2 (1–4)
Respiratory support received			
	No oxygen received	442 (8%)	455 (8%)
	Oxygen only‡	5051 (87%)	4993 (87%)
	Invasive mechanical ventilation	302 (5%)	315 (5%)
Previous diseases			
	Diabetes	1535 (26%)	1569 (27%)
	Heart disease	1267 (22%)	1309 (23%)
	Chronic lung disease	1385 (24%)	1328 (23%)
	Tuberculosis	20 (<1%)	23 (<1%)
	HIV	17 (<1%)	19 (<1%)
	Severe liver disease§	70 (1%)	72 (1%)
	Severe kidney impairment¶	323 (6%)	293 (5%)
	Any of the above	3203 (55%)	3222 (56%)
SARS-CoV-2 PCR test result			
	Positive	5593 (97%)	5566 (97%)
	Negative	126 (2%)	116 (2%)
	Unknown	76 (1%)	81 (1%)
Patient SARS-CoV-2 antibody test result			
	Positive	3078 (53%)	2810 (49%)
	Negative	2016 (35%)	1660 (29%)
	Missing	701 (12%)	1293 (22%)
Corticosteroids received			
	Yes	5370 (93%)	5311 (92%)
	No	391 (7%)	413 (7%)
	Not recorded	34 (1%)	39 (1%)
Other randomised treatments			
	Lopinavir–ritonavir	5 (<1%)	14 (<1%)
	Dexamethasone	3 (<1%)	3 (<1%)
	Hydroxychloroquine	1 (<1%)	0
	Azithromycin	587 (10%)	585 (10%)
	Colchicine	792 (14%)	791 (14%)
	Aspirin	1266 (22%)	1207 (21%)

**Da**ta are mean (SD), n (%), or median (IQR).

* Includes 26 children (<18 years).

† Includes 28 pregnant women.

‡ Includes non-invasive ventilation.

§ Defined as requiring ongoing specialist care.

¶ Defined as estimated glomerular filtration rate <30 mL/min per 1·73 m^2^.

Baseline serology result were available for 9564 (83%) of 11 558 patients, of whom 3676 (38%) were SARS-CoV-2 antibody seronegative (appendix p 51). Patients were more likely to be seronegative if they were older, female, White, had shorter duration of symptoms, were receiving less intensive respiratory support, or were SARS-CoV-2 RNA negative by PCR (appendix p 52). There was an imbalance in the availability of a baseline serology sample, with more missing samples in the usual care group (table 1).

In the convalescent plasma group, 4657 (80%) of 5795 patients received two units, 644 (11%) received one unit, and 494 (9%) received no units (appendix p 53). Two (<1%) patients received both convalescent plasma units from the same donor. In the usual care group, 17 (<1%) of 5763 patients received convalescent plasma. For patients in whom the time of issue of convalescent plasma by the transfusion laboratory was known, 5030 (96%) of 5217 patients had their first unit of plasma issued within 36 h of randomisation. Use of corticosteroids and remdesivir following randomisation was similar between the two groups (appendix p 53). Slightly fewer patients received tocilizumab or sarilumab in the convalescent plasma group (447 [8%] of 5795) than in the usual care group (589 [10%] of 5763 patients; appendix p 53).

**Figure 2:**
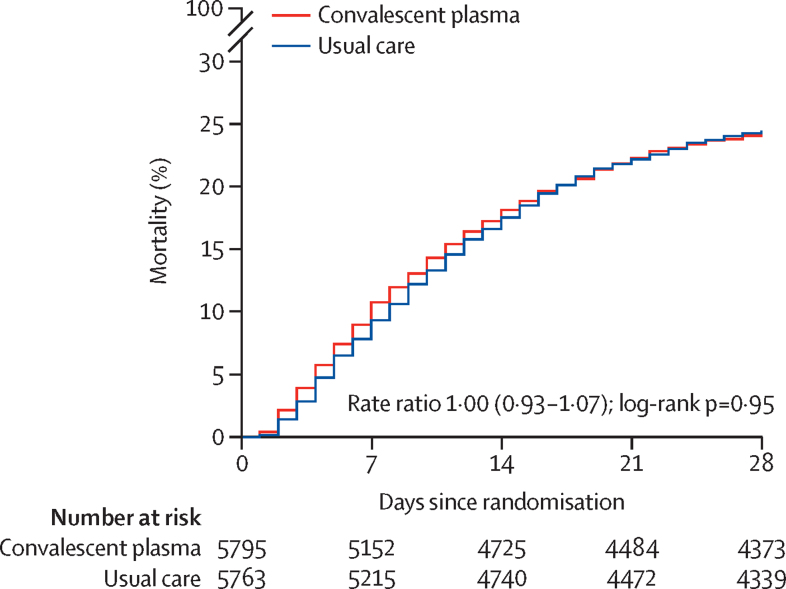
Effect of allocation to convalescent plasma on 28-day mortality

**Figure 3:**
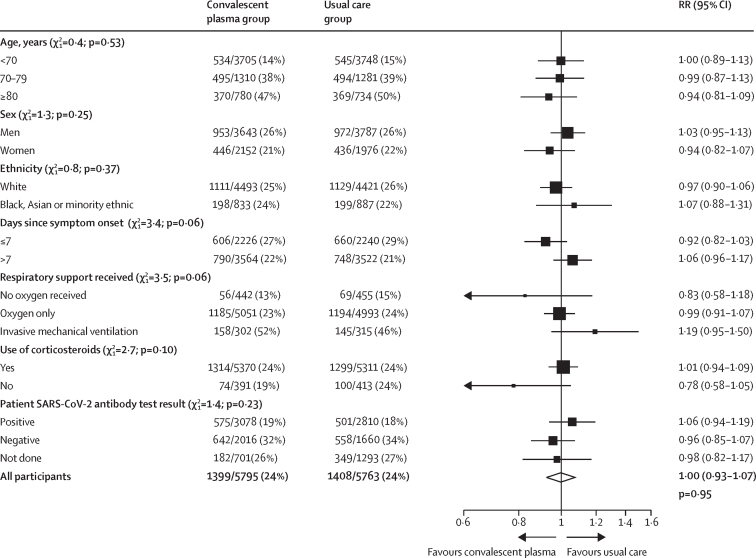
Effect of allocation to convalescent plasma on 28-day mortality by prespecified characteristics at randomisation The ethnicity, days since onset, and use of corticosteroids subgroups exclude those with missing data, but these patients are included in the overall summary. Information on use of corticosteroids was collected from June 18, 2020, onwards following announcement of the results of the dexamethasone comparison from the RECOVERY trial. RR=rate ratio.

**Figure 4:**
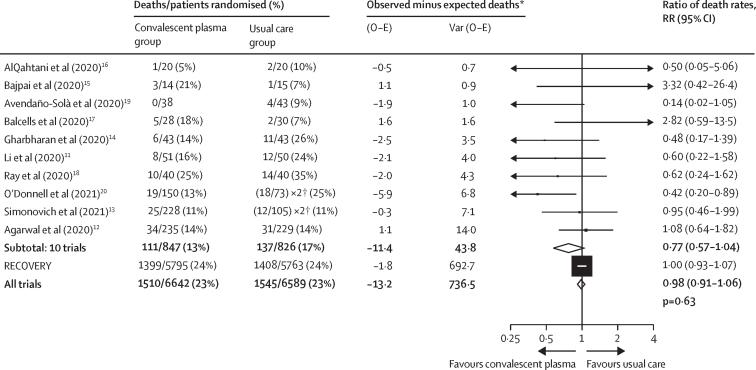
Meta−analysis of mortality in RECOVERY and other trials O–E=observed–expected. Var=variance. RR=rate ratio.*Log−rank O−E for RECOVERY, O−E from 2 × 2 contingency tables for the other trials. RR is calculated by taking ln rate ratio to be (O−E)/V with normal variance 1/V, where V=Var (O–E). Subtotals or totals of (O−E) and of V yield inverse-variance weighted averages of the ln rate ratio values. †For balance, controls in the 2:1 studies count twice in the control totals and subtotals, but do not count twice when calculating their O−E or V values. Heterogeneity between RECOVERY and ten previous trials combined, χ^2^_1_=2·7 (p=0·10).

Primary and secondary outcome data were known for 99% of randomly assigned patients. There was no significant difference in 28-day mortality between the two groups: 1399 (24%) of 5795 patients died in the convalescent plasma group and 1408 (24%) of 5763 patients died in the usual care group (rate ratio 1·00, 95% CI 0·93–1·07; p=0·95; figure 2). We observed similar results across all subgroups with no good evidence of heterogeneity of effect in either the prespecified (figure 3) or the exploratory post-hoc (appendix p 59) subgroup analyses (all p values were >0·05). Results were similar in analyses restricted to patients with a positive SARS-CoV-2 test (rate ratio 1·00, 95% CI 0·93–1·08; p=0·93) and there was no evidence that the rate ratio differed depending on allocation to azithromycin (p>0·1). Although 28-day mortality was higher in patients who were seronegative at randomisation, the proportional effect of allocation to convalescent plasma on 28-day mortality was similar in seropositive patients (575 [19%] of 3078 patients in the convalescent plasma group *vs* 501 [18%] of 2810 patients in the usual care group; rate ratio 1·06, 95% CI 0·94–1·19) and seronegative patients (642 [32%] of 2016 patients in the convalescent plasma group *vs* 558 [34%] of 1660 patients in the usual care group; rate ratio 0·96, 95% CI 0·85–1·07; figure 3; appendix p 60). In ten other reported randomised trials, including a total of 1495 patients hospitalised with COVID-19, 218 of whom died, convalescent plasma was associated with a non-significant reduction in mortality (rate ratio 0·77, 95% CI 0·57–1·04; p=0·08; figure 4).^11^, ^12^, ^13^, ^14^, ^15^, ^16^, ^17^, ^18^, ^19^, ^20^ After inclusion of the results from RECOVERY (which includes nearly eight-times as many patients and more than 11-times as many events as the other trials combined) into this meta-analysis, the mortality rate ratio was 0·98 (95% CI 0·91–1·06; p=0·63; figure 4).

The median time to discharge was 12 days in the convalescent plasma group and 11 days in the usual care group (IQR 6 to >28 in both groups); patients in the convalescent plasma group had a similar probability of being discharge alive within 28 days compared with the usual care group (3832 [66%] of 5795 patients in the convalescent plasma group *vs* 3822 [66%] of 5763 patients in the usual care group; rate ratio 0·99, 95% CI 0·94 to 1·03; p=0·57; table 2). Of the patients who were not receiving invasive mechanical ventilation at baseline, the number of patients progressing to the prespecified composite secondary outcome of invasive mechanical ventilation or death was similar in the two groups (1568 [29%] of 5493 patients in the convalescent plasma group *vs* 1568 [29%] of 5448 patients in the usual care group; rate ratio 0·99, 95% CI 0·93 to 1·05; p=0·79; table 2). For both of these secondary outcomes, there was some evidence of heterogeneity by patient SARS-CoV-2 antibody test result, with slightly more favourable outcomes with convalescent plasma in patients who were seronegative at baseline compared with those who were seropositive (appendix pp 61–62). Because of slight age-imbalances between treatment groups for both seropositive and seronegative patients, an exploratory analysis was done that included adjustment for age, which marginally reduced the apparent heterogeneity (heterogeneity p=0·02 for both secondary outcomes after age adjustment). Results were consistent across all other prespecified subgroups of patients.

**Table 2:** Primary, secondary, and subsidiary outcomes

		**Convalescent plasma group (n=5795)**	**Usual care group (n=5763)**	**RR (95% CI)**	**p value**
**Primary outcome**					
Mortality at 28 days		1399 (24%)	1408 (24%)	1·00 (0·93 to 1·07)	0·95
**Secondary outcomes**					
Median duration of hospitalisation, days		12 (6 to >28)	11 (6 to >28)	..	..
Discharged from hospital within 28 days		3832 (66%)	3822 (66%)	0·99 (0·94 to 1·03)	0·57
Invasive mechanical ventilation or death*		1568/5493 (29%)	1568/5448 (29%)	0·99 (0·93 to 1·05)	0·79
	Invasive mechanical ventilation	678/5493 (12%)	690/5448 (13%)	0·97 (0·88 to 1·08)	0·61
	Death	1241/5493 (23%)	1263/5448 (23%)	0·97 (0·91 to 1·04)	0·46
**Subsidiary outcomes**					
Use of ventilation†		885/3564 (25%)	876/3441 (25%)	0·98 (0·90 to 1·06)	0·55
	Non-invasive ventilation	856/3564 (24%)	845/3441 (25%)	0·98 (0·90 to 1·06)	0·60
	Invasive mechanical ventilation	229/3564 (6%)	238/3441 (7%)	0·93 (0·78 to 1·11)	0·41
Successful cessation of invasive mechanical ventilation‡		85/302 (28%)	108/315 (34%)	0·79 (0·59 to 1·05)	0·11
Renal replacement therapy§		250/5707 (4%)	241/5697 (4%)	1·04 (0·87 to 1·23)	0·69

Data are n (%), median (IQR) or n/N (%). RR=rate ratio for the outcomes of 28-day mortality, hospital discharge, and successful cessation of invasive mechanical ventilation, and risk ratio is calculated for all other outcomes.

* Analyses exclude those on invasive mechanical ventilation at randomisation.

† Analyses exclude those on invasive or non-invasive ventilation at randomisation.

‡ Analyses exclude those not receiving invasive mechanical ventilation at randomisation.

§ Analyses exclude those on renal replacement therapy at randomisation.

There were no significant differences in the prespecified subsidiary clinical outcomes of use of ventilation, successful cessation of invasive mechanical ventilation, or progression to use of renal replacement therapy (table 2), or in cause-specific mortality (appendix p 54).

Within the first 72 h after randomisation, severe allergic reactions were reported in 16 (<1%) of 5795 patients in the convalescent plasma group and two (<1%) of 5763 patients in the usual care group. The frequency of sudden worsening in respiratory status, temperature higher than 39°C or a 2°C or higher increase in temperature above baseline, sudden hypotension, clinical haemolysis, and thrombotic events were broadly similar in the two groups (appendix p 55). We also observed no significant differences in the frequency of major cardiac arrhythmia (appendix p 56). 13 patients receiving convalescent plasma had reports submitted to the Serious Hazards of Transfusion haemovigilence scheme: nine patients with pulmonary reactions (none considered to be transfusion-related acute lung injury, including three deaths possibly related to transfusion), and four patients with serious febrile, allergic, or hypotensive reactions (all of whom recovered).

## Discussion

The results of this large, randomised trial show that convalescent plasma did not improve survival or other clinical outcomes in patients hospitalised with COVID-19. The results were consistent across subgroups of age, sex, ethnicity, duration of symptoms before randomisation, level of respiratory support received at randomisation, and use of corticosteroids. The results are consistent with the evidence from previously reported randomised trials of convalescent plasma for patients hospitalised with COVID-19,^11^, ^12^, ^13^, ^14^, ^15^, ^16^, ^17^, ^18^, ^19^, ^20^ with no evidence of a survival benefit when these results are combined (figure 4).

It has been suggested that the benefits of convalescent plasma depend on the transfused neutralising titre, and that using plasma with lower titres could explain negative results from previous randomised trials. In RECOVERY, all convalescent plasma was supplied via the UK National Blood Services using standardised laboratory processing. Convalescent donors were chosen based on high anti-spike IgG concentrations, using an ELISA that has been shown to correlate well with neutralising antibody.^22^, ^23^, ^24^ We used a EUROIMMUN sample to cutoff ratio of 6·0 for plasma to qualify for use in this trial, which is substantially more than the 3·5 cutoff that the US FDA recognises as high titre.^9^ Nearly all participants received plasma from two different donors to increase the chance that at least one contained higher concentrations of neutralising antibodies.

The presence of anti-SARS-CoV-2 antibodies in recipients at the time of transfusion with convalescent plasma has also been cited as a possible reason for the absence of an observed effect.^14^ In this trial we found that 38% of patients were seronegative at randomisation, and, although they had a markedly higher 28-day mortality risk than patients who were seropositive at randomisation, we did not observe a significant survival benefit from convalescent plasma in these patients. Our results do not exclude the possibility of small improvements in the probability of successful discharge from hospital by day 28 or of progressing to invasive mechanical ventilation or death in seronegative patients who received convalescent plasma. However, the results of these secondary outcomes in one subgroup should be interpreted with caution given the multiple testing; additionally, when an age-adjusted analysis was done the apparent heterogeneity was slightly reduced.

It has also been suggested that antibody-based therapies could be most effective in the early stages of COVID-19, when viral replication dominates.^10^, ^28^ We did not identify a benefit of convalescent plasma when patients were stratified by time since onset of illness in the main analysis or in an exploratory analysis, which subdivided participants on the basis of illness onset. Of note, we did not identify a mortality benefit in the subgroup of patients allocated to convalescent plasma 4 days or less from illness onset, which by itself comprised more patients than the total number of patients enrolled in all other convalescent plasma trials combined. However, RECOVERY only included patients admitted to hospital; therefore, the trial does not address whether convalescent plasma has any benefit if given early after SARS-CoV-2 infection and before the onset of significant disease. That question has not yet been robustly tested in sufficiently large randomised controlled trials.^28^

Following random assignment to receive convalescent plasma, patients with hypoxia and a raised C-reactive protein (≥75mg/L) were eligible for a second random assignment to receive usual care or usual care plus tocilizumab. Although a slightly lower proportion of patients allocated convalescent plasma (8%) subsequently received tocilizumab than patients allocated usual care (10%; appendix p 53), and although tocilizumab itself reduces 28-day mortality by around 15%,^1^ this difference is far too small to have had any material effect on our estimate of the effect of convalescent plasma on mortality (estimated 0·1% difference in 28 day mortality).

SARS-CoV-2 is an RNA virus with antigenic variability. The efficacy of convalescent plasma is likely to depend on the match between the strain-specific transfused anti-SARS-CoV-2 antibodies in donor plasma and the infecting virus variant in the recipient. In December, 2020, a new SARS-CoV-2 variant (B.1.1.7) was detected in the southeast and east of England, with an earliest date of detection in September, 2020. B.1.1.7 spread rapidly to become the dominant SARS-CoV-2 variant, in most regions of the UK, by January, 2021.^29^ Although B.1.1.7 has changes in the spike glycoprotein that could theoretically modify antigenicity, only modest reductions in neutralisation by convalescent plasma have been reported.^27^ Consistent with this, we did not identify any evidence of a differential effect of convalescent plasma before and after the emergence of B.1.1.7 in the UK (appendix p 59).

During an epidemic caused by a novel virus, convalescent plasma is an appealing treatment because it might be available within weeks of the outbreak, long before other targeted therapies are available. Consequently, convalescent plasma has been widely used for COVID-19 outside of clinical trials but, until now, there has been insufficient evidence from randomised trials to reliably assess its safety and efficacy.^10^ In RECOVERY, the largest clinical trial of convalescent plasma for any infectious indication, we did not find evidence that high-titre convalescent plasma improved survival or other prespecified clinical outcomes in patients hospitalised with COVID-19. Whether convalescent plasma would benefit other patient groups is unknown and would need to be evaluated in other, adequately powered, randomised clinical trials.

## Data sharing

The protocol, consent form, statistical analysis plan, definition and derivation of clinical characteristics and outcomes, training materials, regulatory documents, and other relevant trial materials are available online. As described in the protocol, the trial steering committee will facilitate the use of the trial data and approval will not be unreasonably withheld. Deidentified participant data will be made available to bona fide researchers registered with an appropriate institution within 3 months of publication. However, the steering committee will need to be satisfied that any proposed publication is of high quality, honours the commitments made to the trial patients in the consent documentation and ethical approvals, and is compliant with relevant legal and regulatory requirements (eg, relating to data protection and privacy). The steering committee will have the right to review and comment on any draft manuscripts before publication. Data will be made available in line with the policy and procedures available online. Those wishing to request access should complete the form available online and emailed to data.access@ndph.ox.ac.uk.

## Declaration of interests

We declare no competing interests.
